# Spatial genomics of AAV vectors reveals mechanism of transcriptional crosstalk that enables targeted delivery of large genetic cargo

**DOI:** 10.1038/s41587-025-02565-4

**Published:** 2025-03-20

**Authors:** Gerard M. Coughlin, Máté Borsos, Bre’Anna H. Barcelona, Nathan Appling, Acacia M. H. Mayfield, Elisha D. Mackey, Rana A. Eser, Cameron R. Jackson, Xinhong Chen, Sripriya Ravindra Kumar, Viviana Gradinaru

**Affiliations:** 1 Division of Biology and Biological Engineering, California Institute of Technology, Pasadena, CA USA; 2 Aligning Science Across Parkinson’s (ASAP) Collaborative Research Network, Chevy Chase, MD USA

**Keywords:** Genetic vectors, Genetic transduction, Molecular imaging, Animal disease models, Genetic engineering

## Abstract

Cell-type-specific regulatory elements such as enhancers can direct expression of recombinant adeno-associated viruses (AAVs) to specific cell types, but this approach is limited by the relatively small packaging capacity of AAVs. In this study, we used spatial genomics to show that transcriptional crosstalk between individual AAV genomes provides a general method for cell-type-specific expression of large cargo by separating distally acting regulatory elements into a second AAV genome. We identified and profiled transcriptional crosstalk in AAV genomes carrying 11 different enhancers active in mouse brain. We developed spatial genomics methods to identify and localize AAV genomes and their concatemeric forms in cultured cells and in tissue, and we demonstrate here that transcriptional crosstalk is dependent upon concatemer formation. Finally, we leveraged transcriptional crosstalk to drive expression of a 3.2-kb Cas9 cargo in a cell-type-specific manner with systemically administered engineered AAVs, and we demonstrate AAV-delivered, minimally invasive, cell-type-specific gene editing in wild-type mice that recapitulates known disease phenotypes.

## Abstract (web summary)

Profiling transcriptional crosstalk between promoters and cell-type-specific enhancers allows large gene cargo delivery.

## Main

Recombinant adeno-associated viruses (AAVs) are versatile tools for transfer of genetic material, capable of transducing both dividing and non-dividing cells, with minimal immunogenicity^1–5^. Maintenance of the AAV genome as circular monomeric or concatemeric episomes provides long-term expression^6–11^. The tropism of AAVs can be altered by modifying residues on the AAV capsid surface, and directed evolution has yielded a toolkit of capsids with diverse tropisms, including variants that can efficiently and broadly transduce target organs after systemic administration^12–24^.

AAV transduction can also be directed through inclusion of regulatory elements, including enhancer sequences mined from the host genome. Advances in single-cell epigenomics and transcriptomics have facilitated identification of cell-type-specific enhancers that, when transplanted into AAV genomes, can drive expression in a cell-type-specific manner^25–35^. Pairing such regulatory elements with engineered capsids offers the potential for genetic access to specific cell types without the need for transgenic driver lines, opening avenues for targeted manipulation in unconventional model organisms and in translational contexts. Integrating enhancer-driven expression with genome editing and manipulation using CRISPR–Cas-based tools^36–38^ can facilitate understanding of gene function in targeted cell types without confounds due to on-target or off-target editing in other cell types.

AAV delivery of enhancer-driven CRISPR–Cas systems is hindered by AAVs’ relatively low packaging capacity of 4.7 kilobases (kb), including the requisite inverted terminal repeats (ITRs), and large size of regulatory elements and CRISPR effector proteins. In the host genome, enhancers and their target gene(s) are often separated by large distances; chromatin looping can bring distal enhancers and promoters closer in proximity^39,40^. Similarly, Duan et al.^41^ demonstrated that a ubiquitous enhancer in one AAV genome can increase expression from another AAV genome when delivered in *trans*. This phenomenon (which we term ‘transcriptional crosstalk’) represents an underexplored approach for delivery of large cargo to specific cell types.

Increased understanding of AAV genome processing and genome–genome interaction at a single-cell level would facilitate implementation of transcriptional crosstalk as a large cargo delivery method. Spatial methods that preserve anatomical context are particularly well suited to this purpose. By bypassing the need to optimize dissociation protocols for different tissues, spatial methods can be easily applied across tissue types, which is of particular importance for profiling systemically administered gene delivery vectors. Furthermore, spatial methods facilitate profiling of even rare cell types (for example, Purkinje cells (PCs), which, in mice, are outnumbered 200:1 by cerebellar granule cells^42^). Although existing techniques to visualize AAV genomes in situ^43,44^ can provide valuable subcellular information about AAV transduction, such methods are not able to specifically detect certain endpoints of AAV genome processing (for example, concatemeric episomes). Askary et al.^45^ recently developed the Zombie method, in which phage polymerase promoters and barcodes are incorporated into the DNA of interest. Phage RNA polymerase added to the fixed tissue transcribes the barcode, yielding RNA transcripts that can be detected through high-sensitivity hybridization chain reaction fluorescence in situ hybridization (HCR-FISH)^46^. These transcripts serve as proxies for the encoding genome. Importantly, the use of enzymatic amplification may enable specific detection of certain AAV genome states in situ.

In the present study, we investigated transcriptional crosstalk, demonstrating its generalizability to a broad array of minimal promoters and cell-type-specific enhancers. Using single-molecule spatial genomics methods based on Zombie, we explored the mechanism of transcriptional crosstalk in vitro and in vivo and demonstrate critical roles for AAV concatemers in facilitating this phenomenon. Finally, we leveraged transcriptional crosstalk to achieve cell-type-specific delivery of a large Cas9 cargo, after systemic injection of an engineered AAV, resulting in targeted genome editing that recapitulates known behavioral phenotypes.

## Results

### Crosstalk between regulatory elements of separate AAV genomes

**Fig. 1:**
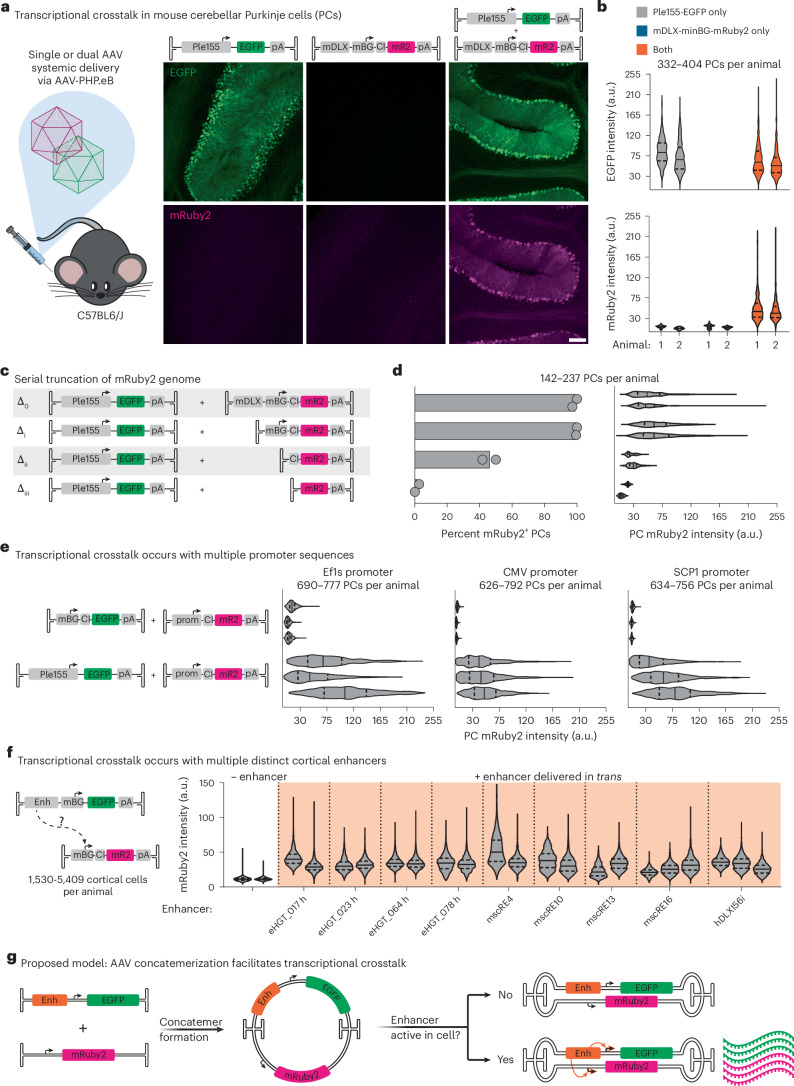
Broad transcriptional crosstalk between enhancers and promoters delivered in separate AAV genomes. **a**, Transcriptional crosstalk. Left column: when injected alone, the AAV-delivered Ple155 element directs strong expression to cerebellar PCs. Middle column: AAV-delivered mDLX-minBG-driven mRuby2 does not yield any detectable PC transduction. Right column: co-administration of both AAVs results in unexpected mRuby2 expression in PCs. All genomes were delivered at 1 × 10^12^ vg dose in AAV-PHP.eB. Scale bar, 100 μm. **b**, Distribution of PC cell body EGFP (top) and mRuby2 (bottom) intensities from animals shown in **a** (*n* = 2 animals per condition). **c**, Schematic of serially truncated mDLX-minBG-mRuby2 constructs, co-injected with Ple155-EGFP, to assess necessity of elements for transcriptional crosstalk. **d**, Quantification of results for truncation conditions shown in **c**, quantified as percent of PCs positive for mRuby2 (left) and PC mRuby2 fluorescence intensity (right). Bars represent mean (*n* = 2 animals per condition). All genomes were delivered at 5 × 10^11^ vg dose in AAV-PHP.eB. **e**, Transcriptional crosstalk between Ple155 and three commonly used minimal promoters (Ef1s, CMV promoter and SCP1) (*n* = 3 animals per condition). All genomes were delivered at 1 × 10^12^ vg dose in AAV-PHP.eB. **f**, Screen of nine cortical enhancers for the ability to upregulate expression of minBG promoter-driven mRuby2 delivered in *trans* (*n* = 2 animals per condition, except mscRE16 and hDLXI56i, in which *n* = 3). All genomes were delivered at 1 × 10^12^ vg dose in AAV-PHP.eB. **g**, Proposed model for transcriptional crosstalk. Formation of concatemeric episomes places enhancer and promoter elements that were delivered in *trans* into a *cis* conformation. This concatemerization facilitates interaction of the enhancer with the promoter that was delivered in *trans*, resulting in increased expression in cells where the enhancer is active. Each violin plot represents data from one animal. CI, chimeric intron.

**Extended Data Fig. 1:**
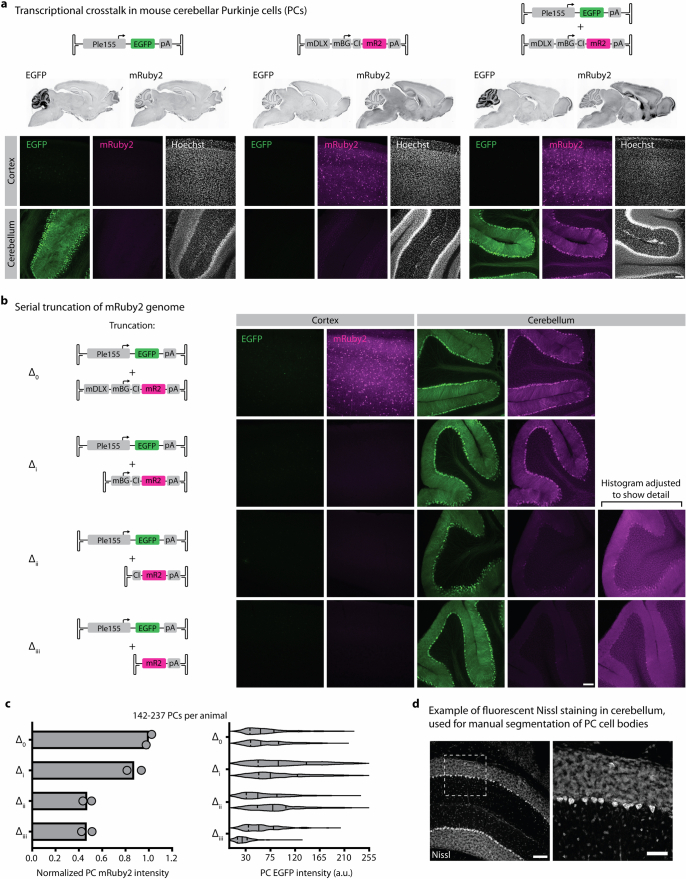
Transcriptional crosstalk in cerebellar PCs between Ple155 and mDLX-minBG. **a**, Related to Fig. 1a,b. Representative images of whole brain, cortical, and cerebellar expression patterns after single or double AAV injections. All genomes delivered at 1e12 vg dose in AAV-PHP.eB. Scale bar = 100 μm. **b**,**c**, Related to Fig. 1c,d. **b**, Representative images of transcriptional crosstalk between Ple155 element and serially-truncated mDLX-minBG. All genomes delivered at 5e11 vg dose, in AAV-PHP.eB. Scale bar = 100 μm. **c**, Quantification of results for truncation conditions shown in (**b**), quantified as normalized mean PC mRuby2 intensity (left) and PC EGFP fluorescence intensity (right). mRuby2 fluorescence intensity is normalized to the mean of full-length mRuby2 genome (construct Δ_0_). Bars represent mean. Each violin plot represents EGFP intensity from PCs in one animal. *n* = 2 animals per condition. All genomes delivered at 5e11 vg dose in AAV-PHP.eB. **d**, Example of fluorescent Nissl staining in cerebellum, showing intense signal in PCs. Nissl staining was used for all manual segmentation of PC cell bodies. Scale bar = 100 μm for left image, 50 μm for right image. CI = chimeric intron.

Transcriptional crosstalk between AAV genomes can occur when regulatory elements in one genome interact with those of another. The Ple155 element^47^ drives strong expression in mouse cerebellar PCs after systemic delivery via a blood–brain barrier (BBB)-penetrant engineered AAV (AAV-PHP.eB^13^). Conversely, the mDLX enhancer^25^ paired with a minimal beta-globin promoter (mDLX-minBG) directs expression to forebrain interneurons but not PCs. However, after co-transduction of these viruses, we observed strong expression of the mDLX-minBG-driven transgene in PCs (Fig. 1a,b and Extended Data Fig. 1a). This result suggests that elements in the Ple155 sequence can interact with elements in the mDLX-minBG genome and increase expression of the latter in a cell-type-specific manner.

To identify which elements in the mDLX-minBG sequence are necessary for this crosstalk, we serially truncated the mDLX-minBG genome (Fig. 1c,d and Extended Data Fig. 1b,c). Removal of the mDLX enhancer did not produce a detectable effect on crosstalk (truncation Δ_i_), whereas removal of the minBG promoter decreased both the percent of mRuby2-positive PCs and the PC mRuby2 intensity (truncations Δ_ii_ and Δ_iii_). These data point to a model in which elements in the Ple155 interact with the minBG promoter, reminiscent of the classical description of enhancer–promoter interaction^39,40^.

**Extended Data Fig. 2:**
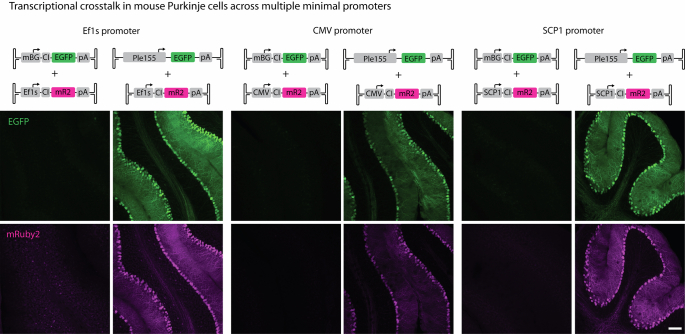
Transcriptional crosstalk occurs across multiple minimal promoters. Related to Fig. 1e. Representative images of transcriptional crosstalk between Ple155 and commonly used minimal promoters: Ef1s (left), CMV promoter (middle), and SCP1 (right). A minBG-driven EGFP serves as a ‘no enhancer’ control. All genomes delivered at 1e12 vg dose in AAV-PHP.eB. Scale bar = 100 μm.

**Extended Data Fig. 3:**
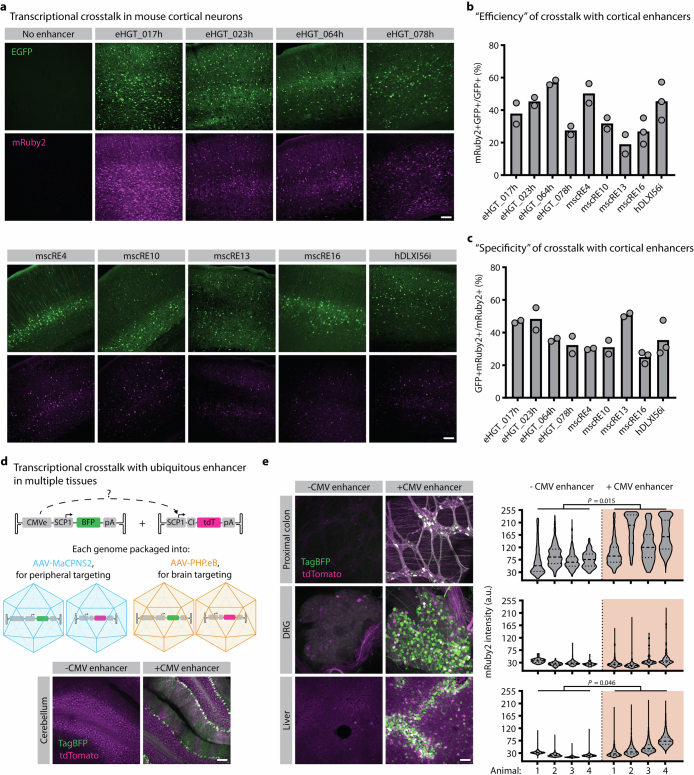
Transcriptional crosstalk in cortex and periphery. **a-c**, Related to Fig. 1f. **a**, Representative images of cortical expression patterns. EGFP reports activity of promoter in *cis* to the enhancer. mRuby2 reports activity of promoter in *trans* to the enhancer. All genomes delivered at 1e12 vg dose in AAV-PHP.eB. Fluorescent protein signal was amplified through IHC. Scale bar = 100 μm. **b**, “Efficiency” of transcriptional crosstalk with various cortical enhancers, quantified as percent of EGFP-positive cells that are also mRuby2-positive. *n* = 2 animals per condition, except mscRE16 and hDLXI56i, for which *n* = 3. **c**, “Specificity” of transcriptional crosstalk with various cortical enhancers, quantified as percent of mRuby2-positive cells that are also EGFP-positive. *n* = 2 animals per condition, except mscRE16 and hDLXI56i, for which *n* = 3. **d**, Strategy for investigating transcriptional crosstalk in multiple tissues. A cocktail of AAV-PHP.eB and AAV-MaCPNS2 was used to provide broad CNS and PNS coverage (1e12 vg per genome-capsid pair, 4e12 vg per animal) and deliver the ubiquitous CMVe and SCP1 promoter. Crosstalk between CMVe and SCP1 was readily apparent in cerebellar PCs (bottom). **e**, Representative images of proximal colon, dorsal root ganglion (DRG), and liver, and quantification of mRuby2 fluorescence intensity. Each violin plot represents mRuby2 intensity from segmented cells in one animal. *n* = 4 animals per condition. Statistical significance was determined using unpaired t-tests. Scale bar = 100 μm. Bars in **b** and **c** represent mean.

Given this model for transcriptional crosstalk, we expect to observe this behavior with multiple promoter and enhancer sequences. Indeed, we observed robust crosstalk between the Ple155 element and three commonly used minimal promoters: Ef1s, the cytomegalovirus (CMV) promoter and the super core promoter 1 (SCP1)^48^ (Fig. 1e and Extended Data Fig. 2). Furthermore, we screened a panel of nine characterized cortical enhancer sequences^25,27,28^, using a minBG-driven mRuby2 crosstalk reporter virus (Fig. 1f and Extended Data Fig. 3a–c). In all nine cases, presence of the enhancer resulted in an increase in expression from the reporter genome delivered in *trans* when compared to a ‘no enhancer’ condition (Fig. 1f).

To further demonstrate the generalized nature of transcriptional crosstalk, we used the ubiquitous cytomegalovirus immediate-early enhancer^49^ (CMVe) and SCP1 in combination with a cocktail of BBB-penetrant and peripheral nervous system tropic engineered AAV capsids (AAV-PHP.eB and AAV-MaCPNS2 (ref. ^16^)) to provide broad central and peripheral nervous system coverage (Extended Data Fig. 3d,e). We observed increased tdTomato crosstalk reporter expression in cerebellum, proximal colon, dorsal root ganglia (DRG) and liver with an enhancer delivered in *trans* versus the ‘no enhancer’ condition.

These results support a generalized model for transcriptional crosstalk, in which enhancer elements on one AAV genome can interact with and drive expression from a promoter on another AAV genome. As this interaction is more likely to occur between elements in *cis*, we and others^41^ propose that concatemerization of AAVs could enable transcriptional crosstalk, by placing elements delivered in *trans* into a *cis* conformation (Fig. 1g).

### AAV-Zombie reveals intracellular AAV genome localization

**Fig. 2:**
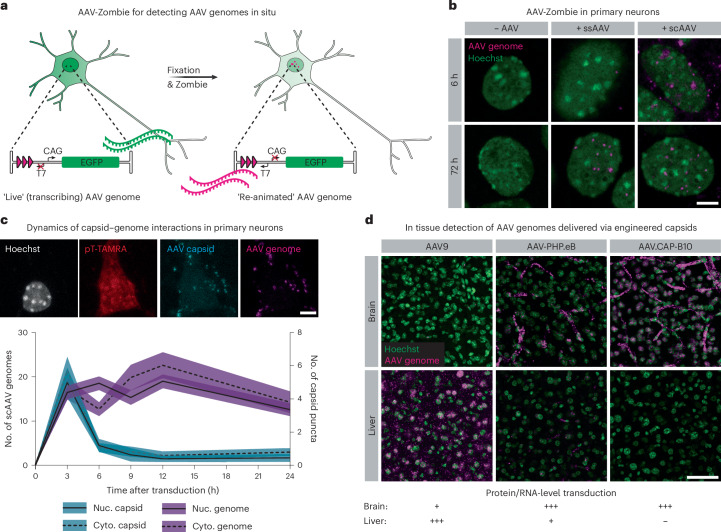
AAV-Zombie reveals intracellular AAV genome localization in cultured cells and in tissue. **a**, Schematic of AAV-Zombie. A barcode and phage RNA polymerase promoter are integrated into the AAV genome. While the cell is alive, the barcode is not transcribed. After fixation, in situ transcription of the barcode by phage RNA polymerase yields barcoded transcripts that can be detected by HCR-FISH. These transcripts serve as a proxy for the AAV genome. **b**, Detection of ssAAV and scAAV genomes in cultured primary neurons. At 6 h after transduction, ssAAV genomes are rarely detected due to the necessity of second strand synthesis, whereas scAAV genomes are readily detected in and outside the nucleus. At 72 h, genomes of both formats are detected in the nucleus. All genomes were delivered at 1 × 10^5^ MOI in AAV6. Scale bar, 5 μm. **c**, Timecourse of AAV capsids and scAAV genomes in nucleus and cytoplasm of primary neurons. Capsids were detected through immunofluorescence with an antibody against linear epitopes. Cytoplasm was labeled with a TAMRA-conjugated polyT probe. Genomes were delivered at 1 × 10^6^ MOI in AAV-DJ. Black line is mean; shaded area is 95% confidence interval (*n* = 243 (*t* = 0 h), 191 (3 h), 317 (6 h), 212 (9 h), 220 (12 h), 255 (24 h) neurons per timepoint). Scale bar, 5 μm. **d**, AAV-Zombie detection of scAAV genomes in C57BL/6J mouse brain and liver 1 d after injection, after systemic delivery by AAV9, AAV-PHP.eB or AAV.CAP-B10, at 3 × 10^11^ vg dose. Distribution of AAV genomes recapitulates known protein-level and RNA-level transduction profiles (bottom). Representative images are from *n* = 3 animals per condition. Scale bar, 50 μm.

To further explore transcriptional crosstalk, we required methods for single-molecule AAV genome localization in intact cells and tissue. We, therefore, adapted the Zombie method^45^ by incorporating phage RNA polymerase promoters and barcodes into the AAV genome (Fig. 2a). In situ transcription and HCR-FISH against the nascent barcoded transcript allow for subcellular localization of both single-stranded AAV (ssAAV) and self-complementary AAV (scAAV) genomes (Fig. 2b). Notably, fixation by methanol and acetic acid is sufficient to release the AAV genome, enabling detection of scAAV genomes irrespective of processing by the host cell (Supplementary Data Fig. 1).

**Extended Data Fig. 4:**
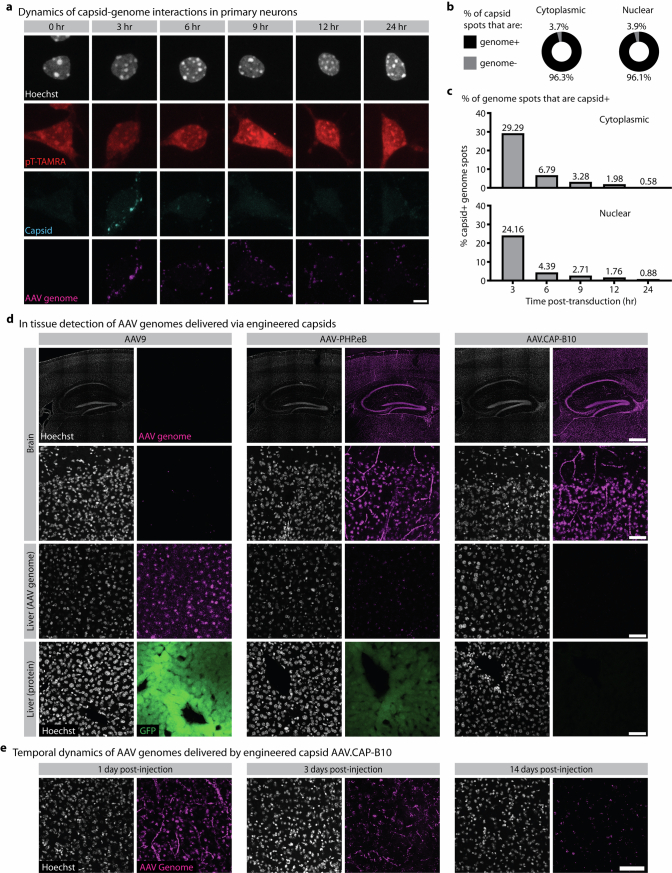
Application of AAV-Zombie to understand AAV transduction *in vitro* and *in vivo.* **a-c**, Related to Fig. 2c. **a**, Representative images of AAV capsids and scAAV genomes, over 24 hrs post-transduction. *n* = 243 (t = 0 hr), 191 (3 hr), 317 (6 hr), 212 (9 hr), 220 (12 hr), 255 (24 hr) neurons per time point. Scale bar = 5 μm. **b**, Percent of capsid puncta that overlap with a scAAV genome, quantified from capsid puncta identified at all time points. The high overlap presumably reflects high encapsidation of the genome. *n* = 763 (nuclear), 1133 (cytoplasmic) capsid puncta. **c**, Percent of genome puncta that overlap with a capsid punctum, for cytoplasmic and nuclear fractions. The decrease in overlap over time reflects uncoating of the AAV genome and degradation of the capsid. For the cytoplasmic fraction, *n* = 3305 (t = 3 hr), 4022 (6 hr), 4201 (9 hr), 5092 (12 hr), 3593 (24 hr) AAV genomes. For the nuclear fraction, *n* = 2421 (t = 3 hr), 3987 (6 hr), 2512 (9 hr), 3469 (12 hr), 2279 (24 hr) AAV genomes. **d**, Related to Fig. 2d. Top 3 rows: AAV-Zombie detection of scAAV genomes in C57BL/6 J brain and liver, following delivery by engineered capsids AAV-PHP.eB and AAV.CAP-B10 and compared to parent capsid AAV9. Tissue was collected 1 day post-injection. Bottom row: EGFP protein in liver, following 2 weeks of expression. Data shows that reduced liver protein expression with AAV-PHP.eB and AAV.CAP-B10 is due to reduced DNA-level transduction, rather than a transcriptional or post-transcriptional difference. Genomes were delivered at 3e11 vg dose. Representative images from *n* = 3 animals per condition. Scale bars = 500 μm for top row, 50 μm for rest. **e**, Time course of AAV.CAP-B10-delivered scAAV genomes in mouse cortex, showing decreasing vascular signal over time. Genomes were delivered at 3e11 vg dose. Representative images from *n* = 3 animals per condition. Scale bar = 500 μm.

Understanding AAV trafficking and processing at early stages of transduction can provide invaluable insights into the vector’s biology. To investigate the dynamics of AAV capsid–genome interaction, we paired AAV-Zombie with immunohistochemistry (IHC) and profiled transduction in primary neuron culture over 24 h (Fig. 2c and Extended Data Fig. 4a–c). As expected, capsid puncta were transient, in both the cytoplasm and nucleus, peaking early in transduction and dropping back to baseline by 12 h. scAAV genomes were more stable over time in both compartments. Notably, more than 96% of capsid puncta co-localized with a genome (across all timepoints); the fraction of genome puncta co-localizing with a capsid was lower and decreased over time (Extended Data Fig. 4b,c).

Given these promising results of AAV-Zombie in cultured cells, we then tested its performance in mouse brain and liver, comparing scAAV genome localization at 1 d after injection between two generations of engineered capsids and their parent AAV9 (Fig. 2d and Extended Data Fig. 4d). Consistent with known protein-level and RNA-level transduction patterns^13,15,19^ (Fig. 2d, bottom), AAV9 accumulated in the liver but was rarely observed in the brain, whereas AAV-PHP.eB and AAV.CAP-B10 (ref. ^15^) both strongly localized to the brain, with reduced liver signal for AAV-PHP.eB and no detected liver signal for AAV.CAP-B10. At this early timepoint, both AAV-PHP.eB and AAV.CAP-B10 showed very strong accumulation in brain vasculature. Tracking of AAV.CAP-B10-delivered genomes over time shows a progressive loss in this vascular signal (Extended Data Fig. 4e). These results demonstrate the power of AAV-Zombie for exploring AAV transduction, both in cultured cells and in tissue.

### SpECTr reveals dynamics of AAV concatemerization

**Fig. 3:**
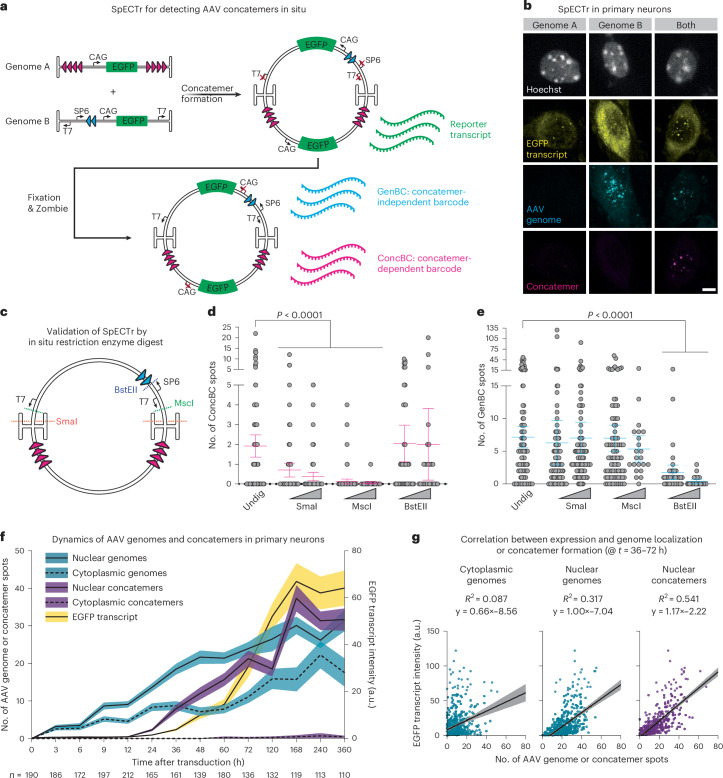
SpECTr reveals spatiotemporal dynamics of AAV concatemerization. **a**, Two AAV genomes are used: Genome A delivers a concatemerization-dependent barcode (ConcBC), and Genome B delivers the T7 RNA polymerase promoter. Concatemerization of these two genomes orients the T7 promoter and the ConcBC such that T7 RNA polymerase can transcribe the ConcBC. Genome B also contains a concatemerization-independent barcode (GenBC), driven by an SP6 RNA polymerase promoter. Both genomes carry a CAG-driven EGFP. **b**, Specificity of SpECTr in primary neurons, 72 h after transduction. Scale bar, 5 μm. **c**–**e**, Validation of SpECTr through in situ restriction enzyme digest of HEK293T cells transduced with SpECTr genomes. **c**, Model AAV concatemer containing one copy of Genome A and one copy of Genome B, showing SmaI, MscI and BstEII restriction enzyme sites. **d**,**e**, Number of ConcBC spots (**d**) and GenBC spots (**e**) detected after in situ restriction enzyme digests, with low (20 U ml^−1^) and high (200 U ml^−1^) restriction enzyme concentrations. Undig, undigested condition in which fixed cells were incubated at 37 °C in restriction enzyme buffer, without any enzyme present. Statistical significance was determined using Kruskal−Wallis test (*P* < 0.0001) with Dunn’s test against the undigested condition. Bars are mean ± s.e.m. (*n* = 138 (Undigested), 99 (low SmaI), 89 (high SmaI), 87 (low MscI), 22 (high MscI), 40 (low BstEII), 26 (high BstEII) cells per condition). **f**, Timecourse of AAV transduction, concatemer formation and EGFP reporter transcription in primary neurons. Cytoplasm was labeled with a TAMRA-conjugated polyT probe, and nucleus was labeled with Hoechst. EGFP transcript intensity was quantified in entire soma; AAV genomes and concatemers were quantified in nucleus and cytoplasm separately. Black line is mean; shaded area is 95% confidence interval. Number of neurons per timepoint is indicated on the figure. **g**, Correlation between EGFP reporter expression and indicated genome states (*n* = 616 primary neurons, pooled from *t* = 36-h, 48-h, 60-h and 72-h timepoints (chosen for detectable EGFP transcript that had not yet plateaued)). Shaded area is 95% confidence interval. For all experiments, genomes were delivered at 1 × 10^6^ MOI in AAV-DJ.

To enable detection of concatemerized AAV genomes, we adapted AAV-Zombie by separating the barcode and T7 RNA polymerase promoter into separate AAV genomes (termed ‘Genome A’ and ‘Genome B’, respectively) (Fig. 3a). Concatemerization of these two genomes orients the T7 promoter and its barcode (hereafter referred to as ‘ConcBC’) such that T7 polymerase can transcribe the barcode. Genome B also contains a barcode (GenBC) driven by an SP6 RNA polymerase promoter, allowing detection of that AAV genome independent of concatemerization. The short length of the phage promoters and barcodes (~20 nucleotides (nt) and 100–250 nt, respectively) leaves ample space for strong mammalian promoters and reporter genes. Thus, after co-transduction, fixation and Zombie, we could detect the concatemer-independent barcode, concatemer-dependent barcode as well as reporter gene transcripts (Fig. 3b), providing single-molecule information about AAV transduction, concatemer formation and expression in single cells. We term this method SpECTr, for ‘SpECTr Enables AAV Concatemer Tracking’.

**Extended Data Fig. 5:**
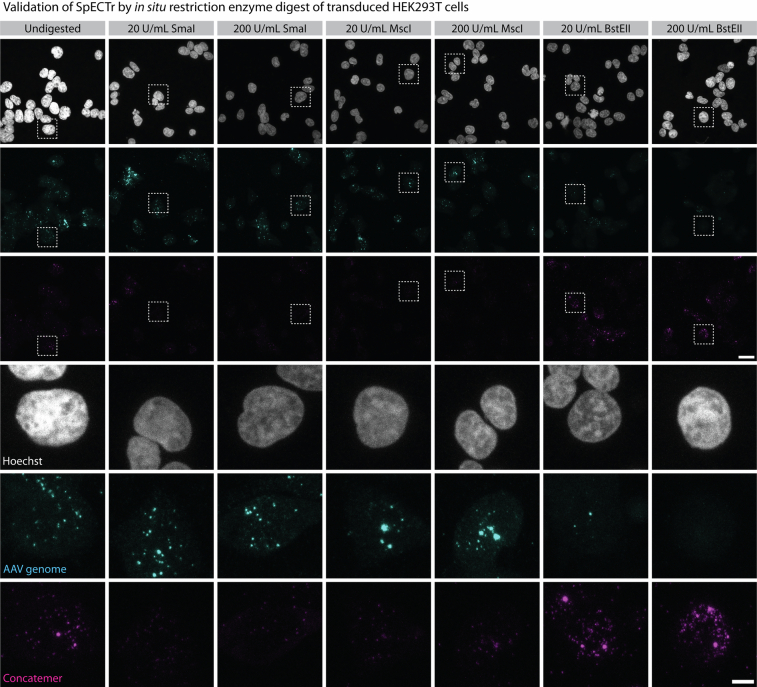
Validation of SpECTr by *in situ* restriction enzyme digest. Related to Fig. 3c-e. Representative images from *in situ* digests of HEK293T cells transduced with SpECTr genomes. “Undig”: undigested condition in which fixed cells were incubated at 37 °C in restriction enzyme buffer without any restriction enzyme. Genomes delivered at 1e6 MOI in AAV-DJ. Scale bars = 20 μm for top 3 rows, 5 μm for rest.

To confirm that the ConcBC transcript arises from a single molecule containing both the T7 promoter and ConcBC, we performed in situ restriction enzyme digests on AAV-DJ-transduced and fixed HEK293T cells before barcode transcription. Digestion with SmaI (which cuts within the AAV ITR) or MscI (which cuts immediately downstream of the T7 promoter) significantly reduced the number of detected ConcBC spots, without affecting the number of GenBC spots. Conversely, digestion with BstEII (which cuts immediately downstream of the SP6 promoter) significantly reduced the number of GenBC spots without affecting the number of ConcBC spots (Fig. 3c–e and Extended Data Fig. 5). These results provided confidence that SpECTr specifically detects AAV concatemers in situ.

**Extended Data Fig. 6:**
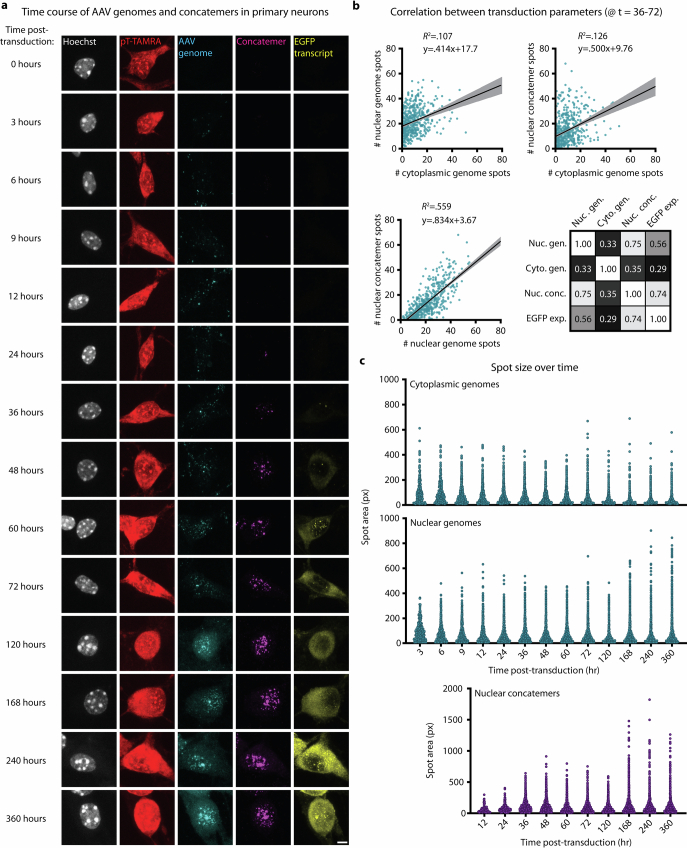
Time course of AAV transduction, concatemerization, and expression in primary neurons. **a-c**, Related to Fig. 3f,g. **a**, Representative images from time course of AAV transduction, concatemer formation, and EGFP reporter transcription in primary neurons. TAMRA-conjugated polyT probe (pT-TAMRA) was used to label cell bodies. Scale bar = 5 μm. **b**, Linear correlations between cytoplasmic AAV genomes, nuclear AAV genomes, and nuclear concatemers, and summary of correlation coefficients for all correlations measured. *n* = 616 primary neurons, pooled from t = 36, 48, 60, and 72 hr time points (time points chosen for detectable EGFP transcript that has not yet reached a plateau). Shaded area is 95% confidence interval. **c**, Distribution of spot sizes for cytoplasmic genomes (top), nuclear genomes (middle), and nuclear concatemers (bottom) over time. *n* = 476 - 2098 (cytoplasmic genomes), 657-4078 (nuclear genomes), 111-4226 (nuclear concatemers) spots per time point.

To test the utility of SpECTr for exploring AAV transduction, we conducted a timecourse of AAV-DJ transduction in primary neurons, collecting samples at 14 timepoints over 360 h after transduction (Fig. 3f and Extended Data Fig. 6a). As expected, we observed an immediate and steadily increasing count of AAV genomes in both the nucleus and cytoplasm. Nuclear concatemeric genome counts began to rise between 12 h and 24 h after transduction, followed shortly after by enhanced green fluorescent protein (EGFP) transcript intensity. The relative order of these increases (genomes, concatemers, transcript) further supports that SpECTr is detecting AAV concatemers. Consistent with specific detection of AAV concatemers, cytoplasmic concatemer counts were low at all timepoints measured (mean < 1 and median = 0, per cell, for each timepoint).

SpECTr provides subcellular and multiparametric data about AAV transduction, enabling us to explore relationships between genome forms, their localization and expression at the single-cell level (Fig. 3g and Extended Data Fig. 6b). Notably, we observed a weak correlation of reporter transcript intensity with cytoplasmic genome counts (*R*^2^ = .087), a moderate correlation with nuclear genome counts (*R*^2^ = .317) and a strong correlation with nuclear concatemer counts (*R*^2^ = .541) (Fig. 3g).

Previous work demonstrated that AAV concatemers can increase in size over time^8,11^. Likewise, we observed variation in the measured concatemer spot area over time (Extended Data Fig. 6c), with larger spots more frequently observed at later timepoints. To assess whether the spot area is indeed related to the size of the concatemer, we transfected HEK293T cells with plasmids containing increasing numbers of T7-barcode repeats and performed Zombie (Supplementary Data Fig. 2). As expected, plasmids with more T7-barcode repeats yielded larger spots.

These results establish AAV-Zombie and SpECTr as validated tools for visualizing AAV genomes and concatemers in situ.

### Reducing AAV concatemer formation decreases crosstalk

**Extended Data Fig. 7:**
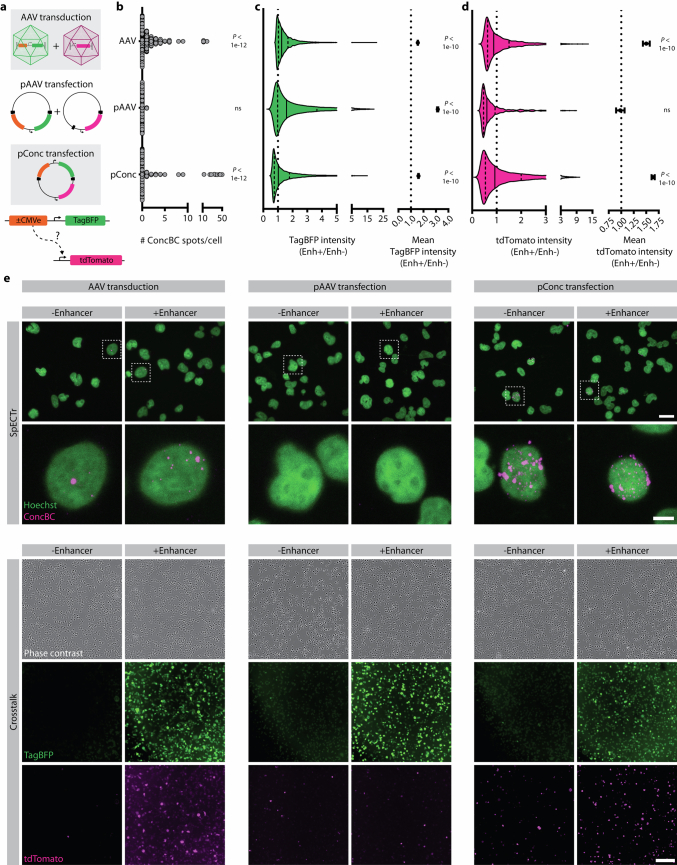
*In vitro* exploration of transcriptional crosstalk mechanisms. **a**, Schematic of experiment. HEK293T cells were either transduced with two cross-talking AAV genomes (top), transfected with two packaging plasmids (middle), or transfected with a single plasmid concatemer (pConc) containing both genomes in *cis* (bottom). For each delivery method, a ‘+ CMV enhancer’ and a ‘no enhancer’ condition were tested. Genomes also contained SpECTr elements. **b**, Quantification of number of ConcBC spots per cell. Statistical significance for SpECTr signal was determined using a Wilcoxon signed rank test, against the null hypothesis that spot count = 0. *n* = 190 (AAV transduction), 220 (pAAV transfection), 227 (pConc transfection) HEK293T cells per condition. **c**,**d**, TagBFP intensity (**c**), and tdTomato crosstalk reporter intensity (**d**), represented as distribution of cell intensities (violin plots) as well as mean ± 95% confidence interval. Fluorescent protein intensity is normalized to the mean of the no-enhancer condition. In all conditions, presence of the enhancer increased expression of TagBFP delivered in *cis*. However, presence of the enhancer increased expression of the tdTomato crosstalk reporter only in the AAV transduction and pConc transfection conditions. Statistical significance for fluorescent protein intensity was determined using one sample t-tests, against the null hypothesis that normalized intensity = 1 (shown by dashed line). For TagBFP: *n* = 14334 (AAV transduction), 18773 (pAAV transfection), 11606 (pConc transfection) TagBFP-positive HEK293T cells per condition. For tdTomato: *n* = 2675 (AAV transduction), 696 (pAAV transfection), 11613 (pConc transfection) tdTomato-positive HEK293T cells per condition. ns = not significant. **e**, Representative images for data quantified in (**b-d**), showing SpECTr signal (upper panel) and reporter fluorescence (lower panel). Scale bars = 20 μm for top row, 5 μm for second row, and 100 μm for rest.

Using SpECTr to visualize AAV concatemers, we next explored the mechanistic connection between concatemerization and transcriptional crosstalk. If concatemerization of AAV genomes enables transcriptional crosstalk (Fig. 1g), then we expect reductions in concatemer formation to reduce transcriptional crosstalk. We first tested this hypothesis in HEK293T cells with the ubiquitous CMVe, comparing AAV-DJ transduction to plasmid transfection (Extended Data Fig. 7). As expected, transcriptional crosstalk was apparent after co-transduction by AAVs but not after co-transfection of the corresponding genome plasmids. Transfection of a ‘plasmid concatemer’, consisting of the entire tdTomato-containing genome inserted outside the ITRs of the TagBFP-containing genome plasmid, recapitulated the co-transduction result. These data suggest that transcriptional crosstalk occurs when the enhancer and promoter are in a *cis* conformation.

We next tested this hypothesis in vivo. Previous research implicated DNA repair pathways in recognizing and processing free ITR ends, resulting in formation of concatemeric AAV episomes^50–56^. In particular, *Prkdc*^*scid/scid*^ mice (hereafter referred to as SCID mice), which have a loss of function in the DNA double-strand break repair enzyme Prkdc, show reduced concatemer formation in bulk muscle^51^ and liver^53^ and lower expression from concatemerization-dependent AAVs^50^. However, neither concatemer formation at a single-cell level nor transcriptional crosstalk has been explored in SCID mice.

**Fig. 4:**
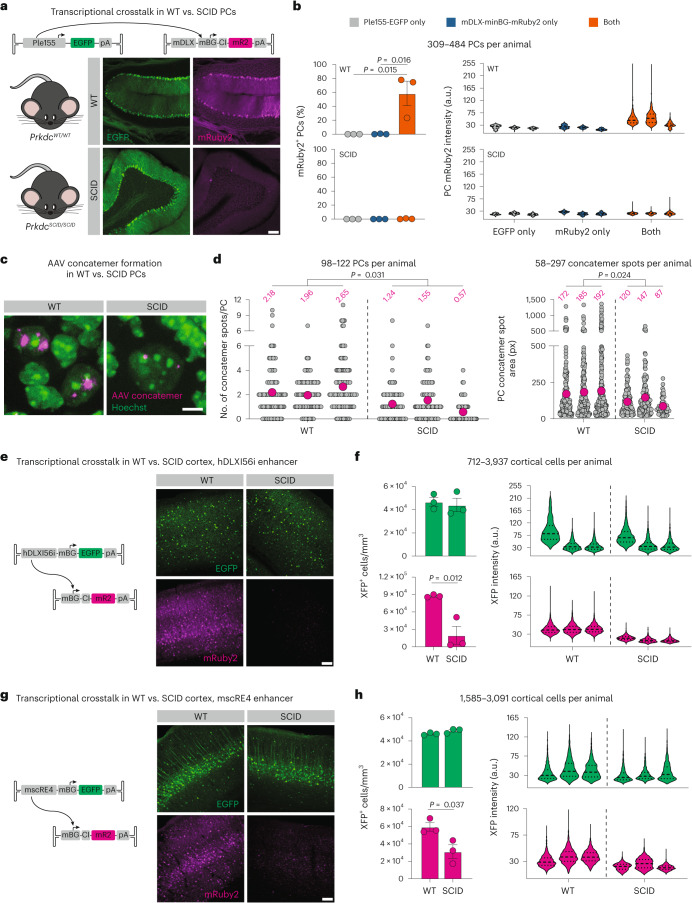
Reducing AAV concatemer formation decreases transcriptional crosstalk between AAV genomes*.* **a**, Representative images of transcriptional crosstalk between Ple155 and minBG promoter, in dual-injected WT and SCID mouse PCs. Both genomes were delivered at 3 × 10^11^ vg dose in AAV-PHP.eB. Scale bar, 100 μm. **b**, Quantification of transcriptional crosstalk shown in **a**, comparing single injection conditions to dual injection condition, and measured as percent of PCs positive for mRuby2 (left) and PC mRuby2 fluorescence intensity (right). Statistical significance was determined using one-way ANOVA (*P* = 0.010) and Tukey’s multiple comparison test (*n* = 3 animals per condition). **c**, Representative images of AAV concatemers detected with SpECTr in PCs of dual-injected WT and SCID animals shown in **a**. Scale bar, 5 μm. **d**, Quantification of PC concatemer spot count (left) and spot size in pixels (px, right), in WT and SCID PCs. Each gray dot corresponds to a single PC (left) or a single concatemer spot (right). Magenta dot and number indicate mean of animal (*n* = 3 animals per condition). **e**–**h**, Representative images and quantification of reduced transcriptional crosstalk in SCID animals with the GABAergic interneuron enhancer hDLXI56i (**e**,**f**) and the layer 5 pyramidal tract neuron enhancer mscRE4 (**g**,**h**). XFP signal was amplified through IHC. Quantification is presented as number of XFP-positive cells per mm^3^ and XFP fluorescence intensity (*n* = 3 animals per condition). Scale bars, 100 μm. Bars in **b**, **f** and **h** represent mean ± s.e.m. Statistical significance in **d**, **f** and **h** was determined using unpaired *t*-tests.

**Extended Data Fig. 8:**
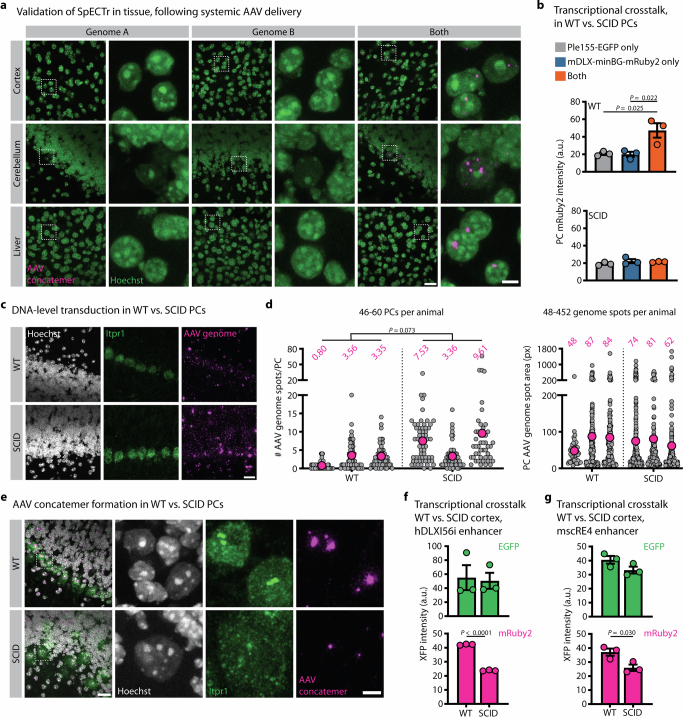
AAV concatemer formation and transcriptional crosstalk in WT and SCID animals. **a**, Validation of SpECTr for detecting AAV concatemers in tissue, using same genomes as in Fig. 3a,b. Wildtype C57BL/6 J mice were transduced with only Genome A (left 2 columns), only Genome B (middle), or both genomes (right). AAV concatemers were nuclear and only detected with SpECTr following co-transduction of both genomes. Scale bars = 20 μm for left images of pairs, 5 μm for right. All genomes delivered at 3e11 vg dose in AAV-PHP.eB. **b**, Related to Fig. 4a,b. Quantification of transcriptional crosstalk in WT and SCID PCs, quantified as mean PC mRuby2 fluorescence intensity. Statistical significance was determined using one-way ANOVA (*P* = 0.015) and Tukey’s multiple comparison test. *n* = 3 animals per condition. Bars represent mean ± s.e.m. **c-e**, Related to Fig. 4c,d. **c**, Representative images of AAV genomes detected and quantified with AAV-Zombie in PCs of dual AAV-injected WT and SCID animals shown in Fig. 4a. HCR-FISH against *Itpr1* transcript serves as a marker for PCs. Scale bar = 20 μm. **d**, Quantification of PC AAV genome spot count (left) and spot size (right), in WT and SCID PCs. Each grey dot corresponds to a single PC (left) or a single genome spot (right). Magenta dot and number indicate mean of animal. *n* = 3 animals per condition. Statistical significance was determined using unpaired t-tests. **e**, Representative images of AAV concatemers detected with SpECTr in PCs of dual-injected WT and SCID animals shown in Fig. 4a. HCR-FISH against *Itpr1* transcript serves as a marker for PCs. Scale bars = 20 μm for left column, 5 μm for others. **f,g**, Related to Fig. 4e-h. Quantification of transcriptional crosstalk in WT and SCID cortical cells, quantified as mean XFP fluorescence intensity, with hDLXI56i enhancer (**f**) and mscRE4 enhancer (**g**). *n* = 3 animals per condition. Bars in (**f**), and (**g**) represent mean ± s.e.m. Statistical significance was determined using unpaired t-tests.

We first validated SpECTr for detection of AAV concatemers in tissues, including cortex, liver and cerebellum (Extended Data Fig. 8a). Then, to enable paired measurement of concatemer formation and transcriptional crosstalk in the same animals, we integrated SpECTr components into the Ple155 and mDLX-minBG AAV genome pair. Reasoning that the high doses of AAVs we used previously (1 × 10^12^ vector genomes (vg); Fig. 1a) may yield many large indistinguishable spots and, thus, confound accurate measurement of concatemers, we injected 3 × 10^11^ total vg into C57BL/6J-background SCID mice and wild-type (WT) C57BL/6J controls. Even at this reduced dose, transcriptional crosstalk was readily apparent in PCs of WT animals; transduction with both genomes resulted in significantly more mRuby2-positive PCs and a significant increase in PC mRuby2 intensity compared to either single transduction condition (Fig. 4a,b and Extended Data Fig. 8b). These effects were not observed in SCID mice.

To determine whether SCID PCs were deficient in AAV concatemerization, we applied AAV-Zombie and SpECTr to cerebellum sections from the same animals. PCs were identified using HCR-FISH against *Itpr1* transcript^57^. With AAV-Zombie, we measured a 2.6-fold higher AAV genome count in SCID than WT PCs (Extended Data Fig. 8c,d). In a separate cohort of mice, we similarly observed significantly higher DNA-level transduction of SCID brains by AAV-PHP.eB, with no significant differences in protein-level transduction (Supplementary Data Fig. 3). Despite higher DNA-level transduction of SCID brains, SpECTr revealed significantly fewer and smaller ConcBC spots in PCs of SCID mice than WT controls (Fig. 4c,d and Extended Data Fig. 8e), indicating reduced concatemer formation in the absence of functional Prkdc.

Finally, we assessed whether the SCID mutation would affect crosstalk of other enhancers as well. We chose two additional enhancers, targeting GABAergic interneurons (hDLXI56i)^25^ and layer 5 pyramidal tract excitatory neurons (mscRE4)^27^, and co-injected these with an mRuby2 crosstalk reporter (Fig. 4e–h and Extended Data Fig. 8f,g). Consistent with our observations from the Ple155 and mDLX-minBG pair, we observed reduced transcriptional crosstalk with both enhancers in SCID mice, quantified by both number of mRuby2-positive cells per mm^3^ and fluorescence intensity of mRuby2-positive cells. We did not detect any difference in transduction between genotypes, as assessed by number and intensity of EGFP-positive cells.

Taken together, these in vitro and in vivo results strongly suggest that AAV concatemer formation enables transcriptional crosstalk. As concatemer formation appears to be a common endpoint of AAV genome processing, and given the generalizability of the phenomenon across cell-type-specific enhancers, we next explored whether we could leverage transcriptional crosstalk to achieve cell-type-specific expression of large cargos.

### Crosstalk enables all-AAV cell-type-specific gene editing

**Fig. 5:**
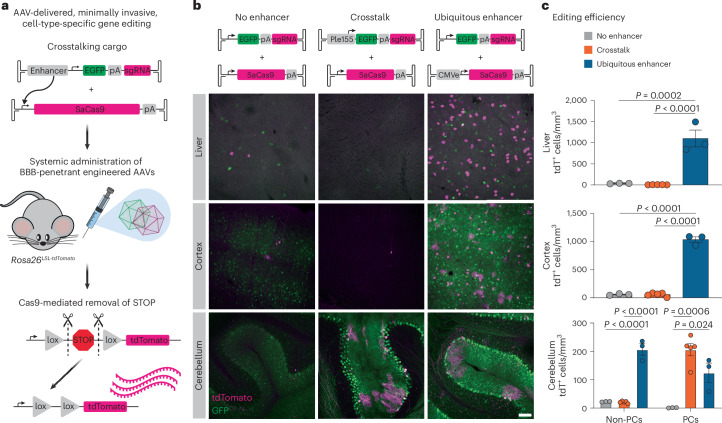
Transcriptional crosstalk enables all-AAV cell-type-specific genome editing with CRISPR–Cas9. **a**, Schematic of AAV-delivered, minimally invasive, cell-type-specific gene editing. SaCas9, packaged with minimal elements (total size, 4.2 kb), is delivered with a bulky enhancer element in *trans*, resulting in upregulation of SaCas9 expression in a cell-type-specific manner. As a proof of principle, we used a common reporter assay with *Rosa26*^*LSL-tdTomato*^ mice, in which guide RNAs direct SaCas9 to remove the stop cassette, enabling tdTomato expression. For all conditions, the sgRNAs were expressed by the ubiquitous U6 promoter. All genomes were delivered at 1 × 10^12^ vg dose in AAV-PHP.eB. **b**, Demonstration of crosstalk-enabled gene editing using the Ple155 element to drive SaCas9 expression in PCs. As controls, we included a ‘no enhancer’ condition as well as a condition in which SaCas9 is strongly expressed by the ubiquitous CMVe delivered in *cis*. Representative images are from liver, cortex and cerebellum. Scale bars, 100 μm. **c**, Quantification of editing efficiency, assessed by number of tdTomato-positive cells per mm^3^ of tissue. PCs and non-PCs were quantified separately. Using crosstalk to drive strong SaCas9 expression specifically in PCs restricted high-efficiency editing to that cell type. Statistical significance was determined using one-way ANOVAs (for liver, cortex and non-PCs, *P* < 0.0001; for PCs, *P* = 0.0008) and Tukey’s multiple comparison test (*n* = 3 (no enhancer or ubiquitous enhancer) or *n* = 5 (crosstalk) animals per condition). Bars represent mean ± s.e.m.

We reasoned that transcriptional crosstalk might enable cell-type-specific delivery of larger cargo, by separating bulky gene regulatory elements from minimal promoters and coding sequences in another AAV (Fig. 5a). We explored the feasibility of this approach using *Staphylococcus aureus* Cas9 (SaCas9) as a large cargo and targeting PCs with Ple155. Notably, these two sequences (3.2 kb and 1.65 kb, respectively) are too large to fit into a single AAV genome together. We adopted a commonly used reporter assay based on Ai14 mice (*Rosa26*^*CAG-LSL-tdTomato*^)^58–61^.

Minimal expression of SaCas9 with no enhancer resulted in low efficiency of editing in all tissues examined (Fig. 5b,c, ‘no enhancer’). Conversely, when SaCas9 was strongly expressed with a ubiquitous enhancer (CMVe) delivered in *cis*, we observed a strong increase in editing efficiency in tissues of interest compared to the no-enhancer condition. We saw a 31-fold increase in editing in the liver, a 17-fold increase in the cortex, a nine-fold increase in non-PCs and a 107-fold increase in PCs (Fig. 5b,c, ‘ubiquitous enhancer’). Using transcriptional crosstalk to direct SaCas9 expression specifically to PCs with the Ple155 element in the companion AAV genome, we restricted efficient editing to PCs, yielding a 177-fold increase in PC editing efficiency compared to the no-enhancer condition, with no significant increases in other tissues and cerebellar cell types (Fig. 5b,c, ‘crosstalk’). These results establish the utility of transcriptional crosstalk for AAV-based cell-type-specific genome editing and manipulation, bypassing the need for transgenic driver lines to restrict expression to a target population or Cas9 reporter lines to deliver editing machinery.

### Harnessing crosstalk for cell-targeted functional genetics

Efficient and specific gene editing through transcriptional crosstalk from systemically delivered AAVs offers a means to explore gene function in a cell-type-specific manner. Notably, this strategy does not rely on transgenic lines, enabling rapid and cost-effective generation of large cohorts from easily obtained WT animals.

**Fig. 6:**
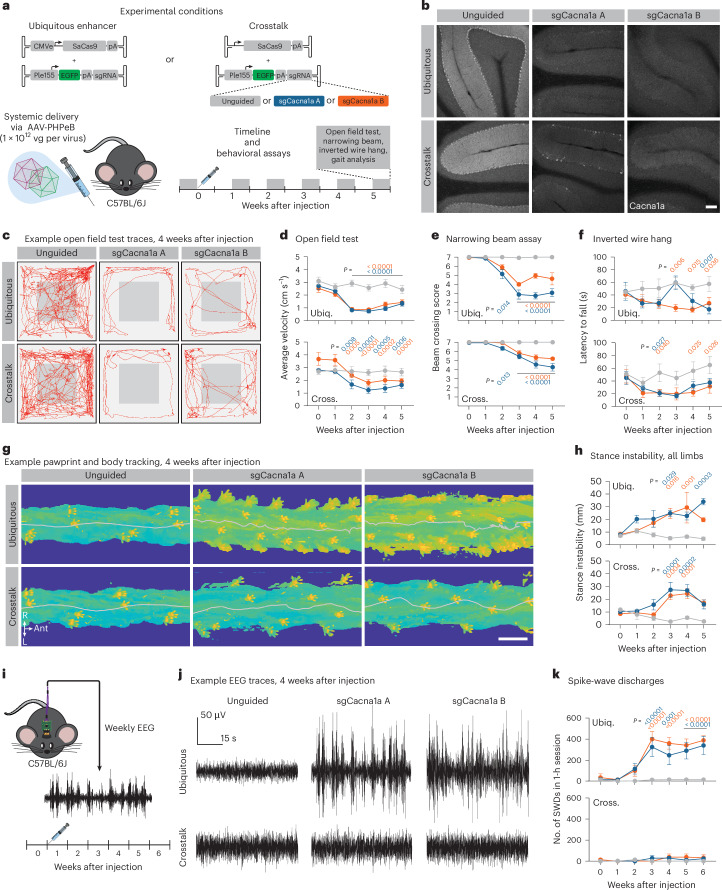
Transcriptional crosstalk enables efficient cell-type-specific gene disruption. **a**, Two conditions were tested. In the ubiquitous condition, SaCas9 was strongly expressed with CMVe delivered in *cis*. In the crosstalk condition, SaCas9 expression was restricted to PCs through inclusion of the Ple155 element delivered in *trans*. Two sequence-independent sgRNAs targeting *Cacna1a* were used and compared to an unguided condition. sgRNAs were expressed by the ubiquitous U6 promoter. Behavioral assays were performed weekly, before and 5 weeks after AAV administration. **b**, Representative IHC against Cacna1a in cerebellum. Scale bar, 100 μm. **c**–**h**, Characterization of ataxic phenotypes after ubiquitous (‘Ubiq.’, top graphs) and PC-specific (‘Cross.’, bottom graphs) disruption of *Cacna1a*. Motor behavior was assessed with an open field test (**c**,**d**), skilled locomotion with the narrowing beam assay (**e**), limb strength with inverted wire hang (**f**) and gait using automated pawprint and body tracking (**g**,**h**). Red lines in **c** represent animal position over a 10-min trial, 4 weeks after injection. Heatmaps in **g** show pawprint positions and body tracking over a small segment of the elevated platform used for gait analysis, 4 weeks after injection. Gray line indicates midline of body. Scale bar, 3 cm. **i**–**k**, Characterization of epileptic activity in cortex for same experimental manipulations as in **a**. **i**, Weekly 90-min EEG recordings were collected, before and for 6 weeks after AAV administration. **j**, Sample cortical EEG traces, 4 weeks after injection. **k**, Quantification of SWDs for animals in ubiquitous (top) and PC-specific (bottom) conditions. Statistical significance for open field test, beam crossing, stance instability and SWDs was determined by two-way repeated-measures ANOVA and Dunnett’s multiple comparison tests against 0-week timepoint. Statistical significance for inverted wire hang was determined by two-way repeated-measures ANOVA and Dunnett’s multiple comparison tests against the unguided condition. Points and bars represent mean ± s.e.m. For all groups, *n* = 5 (except ubiquitous + sgCacna1a B behavior group, crosstalk + sgCacna1a A behavior group and ubiquitous + sgCacna1a B EEG group, in which *n* = 6). Gray line, unguided controls; blue, sgCacna1a A; orange, sgCacna1a B.

To test this approach, we targeted *Cacna1a* in WT C57BL/6J mice, in either a ubiquitous or a PC-specific manner using transcriptional crosstalk (Fig. 6a, top). *Cacna1a* is broadly expressed in the brain, and global knockout leads to dystonia, ataxia, cerebellar degeneration, absence seizures and early lethality^62,63^. Forebrain-specific deletion of *Cacna1a* causes learning and memory deficits^64^ and leads to the emergence of absence seizures^65,66^. PC-targeted loss of function through a Pcp2-cre driver line leads to ataxia^67^ and, unexpectedly, absence seizures^68^. This epileptiform activity was attributed to recombinase activity from this driver line in forebrain populations^68,69^. Thus, understanding the function of *Cacna1a* in PCs requires methods to specifically target PCs, thereby avoiding confounds due to loss of function in other brain regions.

As a control for the effects of off-target editing, we used two sequence-independent guide RNAs (sgCacna1a A and sgCacna1a B), comparing these to an unguided condition in which no guide RNA sequence was present. To assess whether this approach could recapitulate known ataxia phenotypes resulting from PC-specific loss of function of *Cacna1a*, we assessed a battery of behaviors before and for 5 weeks after AAV administration (Fig. 6a, bottom).

**Extended Data Fig. 9:**
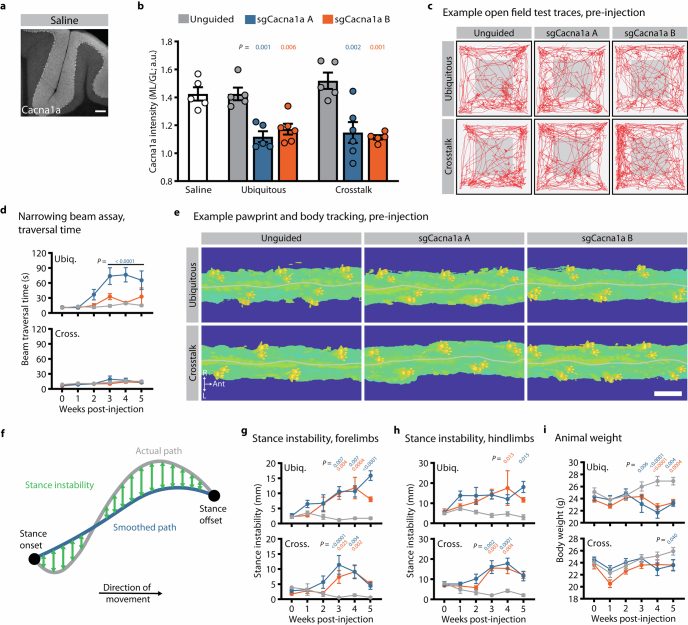
Transcriptional crosstalk-mediated knockout of Cacna1a in Purkinje cells results in ataxic phenotypes. **a**,**b**, Related to Fig. 6b. **a**, Representative image of IHC against Cacna1a in cerebellum of saline-injected control animal. Scale bar = 100 μm. **b**, Quantification of Cacna1a staining in cerebellum, normalized as the intensity in the molecular layer (ML) divided by the intensity in the granular layer (GL). Statistical significance was determined by one-way ANOVA and Dunnett’s multiple comparison tests against Cacna1a intensity in age-matched saline-injected mice. **c**, Related to Fig. 6c,d. Example open field test traces acquired pre-injection, showing same animals as in Fig. 6c. Red lines represent animal position over a 10 min trial. **d**, Related to Fig. 6e. Beam traversal time for narrowing beam assay following ubiquitous and PC-specific disruption of *Cacna1a*. **e-h**, Related to Fig. 6g,h. **e**, Example pre-injection pawprint and body tracking, over a small segment of the elevated plexiglass platform used for gait analysis, showing same animals as in Fig. 6g. Grey line indicates midline of body. Scale bar = 3 cm. **f**, Schematic to demonstrate stance instability metric. For each stance cycle, tracking of body center relative to paw location yields a stance trace (grey line) and a smoothed version of that trace (blue line). The summed difference between the actual path and the smoothed path corresponds to the stance instability. **g**,**h**, Stance instability for forelimbs (**g**) and hindlimbs (**h**) following ubiquitous and PC-specific disruption of *Cacna1a*. **i**, Animal weights following ubiquitous and PC-specific disruption of *Cacna1a*. Statistical significance for beam crossing and stance instability was determined by two-way repeated-measures ANOVA and Dunnett’s multiple comparison tests against behavioural performance at 0-week time point. Statistical significance for animal weight was determined by two-way repeated-measures ANOVA and Dunnett’s multiple comparison tests against the unguided condition. Points and bars represent mean ± s.e.m. For all groups, *n* = 5 except (ubiquitous + sgCacna1a B) and (crosstalk + sgCacna1a A) groups, in which *n* = 6. For all plots, grey line = unguided controls, blue = sgCacna1a A, and orange = sgCacna1a B.

In both ubiquitous and PC-specific paradigms, we observed a strong reduction in Cacna1a staining in the cerebellum that was consistent between both guide RNAs (Fig. 6b and Extended Data Fig. 9a,b). Notably, we saw similar reductions in Cacna1a staining intensity with both ubiquitous and PC-specific SaCas9 expression.

Both ubiquitous and PC-specific *Cacna1a* disruption also recapitulated several hallmarks of ataxia: reduced locomotion in an open field (Fig. 6c,d and Extended Data Fig. 9c); impairments in skilled motor behavior, as assessed by narrowing beam crossing (Fig. 6e, Extended Data Fig. 9d and Supplementary Videos 1 and 2); reduced limb strength (Fig. 6f); and gait deficits (Fig. 6g,h and Extended Data Fig. 9e–h). Both guide RNAs resulted in similar phenotypes, suggesting that the deficits observed were not due to off-target editing, and no deficits were observed in animals that did not receive a guide RNA. Whereas ubiquitous expression of SaCas9 led to significantly reduced weight by 3 weeks after injection, we did not observe any significant difference in weight until 5 weeks after injection with PC-specific expression (Extended Data Fig. 9i).

We next assessed whether the specificity afforded by transcriptional crosstalk in the PC-specific paradigm could circumvent the epileptic activity observed with forebrain-specific disruption of *Cacna1a*. Thus, in a separate cohort of animals, we conducted longitudinal cortical electroencephalography (EEG) recordings, starting 1 week before and continuing 6 weeks after AAV injection (Fig. 6i), monitoring for spike-and-wave discharges (SWDs) that are characteristic of absence seizures.

**Extended Data Fig. 10:**
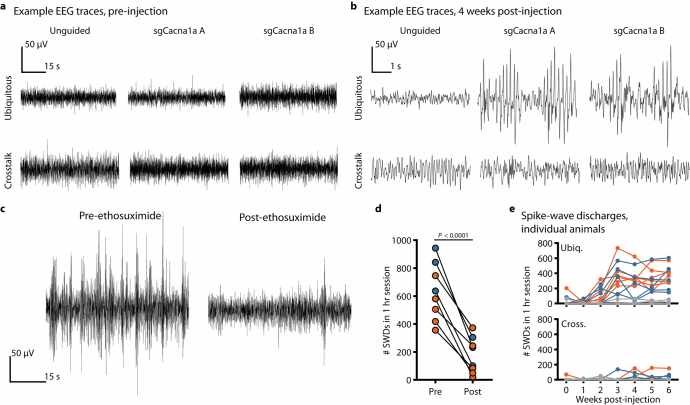
Transcriptional crosstalk-mediated knockout of Cacna1a in Purkinje cells does not result in absence seizures. Related to Fig. 6i-k. **a**, Example cortical EEG traces, acquired pre-injection, showing same animals as in Fig. 6j. **b**, Higher temporal resolution cortical EEG traces, acquired post-injection, corresponding to those shown in Fig. 6j. **c**, Example EEG traces acquired before and after ethosuximide administration. **d**, Number of spike-and-wave discharges (SWDs) detected in 1 hr sessions, before and after ethosuximide administration, in animals from the ubiquitous condition. The observed reduction in the number of detected SWDs provides confidence in our SWD detection and quantification pipeline. Statistical significance was determined using a paired t-test. **e**, Quantification of SWDs for individual animals in ubiquitous (top) and PC-specific conditions (bottom). For all plots, grey line = unguided controls, blue = sgCacna1a A, and orange = sgCacna1a B.

In the ubiquitous paradigm, both single guide RNAs (sgRNAs) against *Cacna1a* resulted in significant increases in detected SWDs by 3 weeks after injection (Fig. 6j,k and Extended Data Fig. 10). This was not observed in the unguided condition. Notably, transcriptional crosstalk-mediated PC-specific *Cacna1a* disruption did not result in a significant increase in detected SWDs. However, in a small number of animals targeted through transcriptional crosstalk, we did detect slight increases in SWDs (Extended Data Fig. 10e), potentially reflecting some leakiness due to basal activity of the minBG promoter.

Taken together, the results of our behavioral and EEG experiments demonstrate that transcriptional crosstalk can be leveraged for cell-type-specific gene manipulation in WT animals.

## Discussion

Diversification of the AAV capsid through directed evolution has yielded a toolkit of AAVs with varied tissue tropism^12–24^. Further refinement of expression can be achieved through inclusion of regulatory elements, including enhancer sequences^25–34^. However, successful incorporation of these elements requires an understanding of how AAV genomes are processed by the host cell, necessitating development of novel methods for visualizing and measuring AAV genomes in situ.

To this end, we developed two complementary methods: AAV-Zombie and SpECTr. AAV-Zombie enables single-molecule visualization of the AAV genome in situ, allowing us to profile capsid–genome interactions in cultured cells and to assess DNA-level transduction of engineered viral vectors in tissue (Fig. 2). Although protein-level and RNA-level measurements of AAV transduction have provided invaluable insights at the single-cell level, these methods can miss cell types that are recalcitrant to AAV genome expression^43^. Furthermore, whereas previous studies detected AAV genomes in situ using long (~700 nt) probes^44^ or an array of probes paneled across the genome^43^, our methods rely only on short barcode sequences (here, as short as two 40-nt barcodes). The use of short barcodes can simplify construction of barcoded libraries that are read out with a sequential FISH paradigm^19^. Such refinements to AAV-Zombie may enable DNA-level profiling of large capsid pools or libraries in situ.

Complementary to AAV-Zombie, SpECTr enables single-molecule visualization of AAV concatemers in situ (Fig. 3). Development and implementation of AAV gene therapies necessitates better understanding of genome processing in cell types relevant to disease, especially given the stability of episomal AAV genomes in host cells and implicated roles of AAV concatemers in productive transduction^56,70^. SpECTr can help address this critical knowledge gap, as we highlight here with our multiparametric single-cell AAV transduction profiling in primary cell cultures.

Concatemerization of AAV genomes can have unintended consequences when delivering multiple genomes. Duan et al.^41^ demonstrated transcriptional crosstalk with a ubiquitous enhancer in cultured cells and in muscle tissue. In the present study, we extend this observation to multiple promoters and cell-type-specific enhancers in a variety of tissue and cell types. We observed transcriptional crosstalk occurring with four separate promoters and all 11 enhancers that we tested, suggesting that this is a general phenomenon (Fig. 1). Using SpECTr, we mechanistically link this transcriptional crosstalk to concatemer formation (Fig. 4).

Transcriptional crosstalk between AAV genomes may lead to undesired expression, especially in cases where multiple enhancers are used for simultaneous targeting of different cell types. For example, pooled AAV screening is used to reduce the number of animals necessary to profile enhancer elements^26,32^. Crosstalk between genomes in such pools may confound the resulting transduction profiles, especially in cases where the number of variants is small (for example, two enhancers, 5 × 10^11^ vg each). Screening large pools at lower multiplicities of infection (MOIs) (for example, 100 enhancers, 1 × 10^10^ vg each) can help to mitigate this issue, by reducing concatemerization of the same genomes in multiple cells. Performing pooled AAV enhancer screens in animals with mutations in DNA repair pathways associated with concatemerization offers another solution to this issue. Our results with SCID mice support this approach (Fig. 4).

Transcriptional crosstalk also presents an opportunity to overcome some of the limitations of AAVs’ small packaging capacity by separating distally acting elements from *cis*-acting elements and coding sequences. We demonstrate this here using Cas9 nuclease, directing editing to specific cell types in vivo (Fig. 5), with sufficient efficiency and coverage of the target population to recapitulate known loss-of-function behavioral phenotypes while avoiding confounding phenotypes due to gene disruption in off-target cells (Fig. 6).

Notably, this paradigm does not require transgenic organisms and so may be easily applied to a variety of models, including disease models where crossing of transgenics would not be feasible as well as models where Cas9-expressing transgenics are not readily available. This approach is particularly exciting given the recent development of AAV capsids that provide genetic access to the nervous systems of non-human primates after systemic administration^16–18,24,71^ and the identification of gene regulatory elements for cell-type-specific expression across species^25,28–30,34,35^. The small size of minimal promoters and terminator sequences allows easy integration of even large CRISPR effectors, including fusion proteins for AAV-based gene activation^72^, base editing^73^ or prime editing^74^. Cell-type-specific delivery of larger cargos could also be achieved through integration of transcriptional crosstalk with existing dual vector strategies, such as mRNA or protein *trans*-splicing^1^. Targeting of specific cell types with these genome editing tools after minimally invasive delivery of crosstalking genomes may open new avenues of research into gene function in a myriad of model organisms.

Transcriptional crosstalk may also have applications in translational contexts. Cell-type-specific delivery of gene editing and manipulation machinery may help to reduce side effects due to on-target or off-target changes in untargeted cell types^75,76^. In addition, transcriptional crosstalk may enable higher or cell-type-specific expression of large therapeutically relevant cargo; some examples of indications where this strategy may be beneficial are listed in Supplementary Table 1. Finally, incorporation of additional regulatory elements, such as microRNA target sites or on-switches^1,19,60^ or combinations of multiple enhancer sequences, could help to further hone expression and/or enable temporal control.

In summary, we identified and profiled transcriptional crosstalk occurring between promoters and cell-type-specific enhancers delivered on separate AAV genomes. We paired transcriptional crosstalk with systemically administered BBB-penetrant AAVs to enable minimally invasive delivery of a large Cas9 cargo for cell-type-specific gene disruption in WT animals, which recapitulated phenotypes of genetic knockouts. To understand the mechanisms underlying transcriptional crosstalk, we developed and validated spatial genomics techniques—AAV-Zombie and SpECTr—that enable tracking of AAV genomes and concatemers in intact cells and tissue. Leveraging these methods, we demonstrated that concatemerization of the AAV genome facilitates transcriptional crosstalk. These novel spatial genomics techniques can help to bridge a critical knowledge gap linking AAV genome processing with expression and enable integration of gene regulatory elements for genetic access to and manipulation of targeted cell populations, both in basic research and in translational contexts.

## Methods

### Key resources

Key resources for this work, including novel plasmids deposited to Addgene, are listed in Supplementary Table 2.

### Plasmid DNA

Standard molecular cloning techniques were used to generate DNA constructs in this study. Double-stranded DNA was synthesized by Integrated DNA Technologies and inserted into pAAV backbones with NEBuilder HiFi (New England Biolabs, E2621). sgRNA sequences were synthesized as overlapping single-stranded DNA oligos (Integrated DNA Technologies) that were then annealed together and ligated into sgRNA expression cassettes using T4 DNA ligase (New England Biolabs, M0202). Plasmids used in Supplementary Data Fig. 2 were constructed from polymerase chain reaction (PCR)-amplified DNA fragments (Integrated DNA Technologies) assembled via Golden Gate Assembly (New England Biolabs, E1602S). Sequences of sgRNAs and utilized DNA elements (for example, promoters and enhancers) are provided in Supplementary Table 3. Self-complementary pAAVs were generated from pscAAV-CAG-GFP, a gift from Mark Kay (Addgene, 83279).

pUCmini-iCAP-AAV-PHP.eB^13^ (Addgene, 103005), pUCmini-iCAP-AAV.CAP-B10 (ref. ^15^) (Addgene, 175004), pUCmini-iCAP-AAV.MaCPNS2 (ref. ^16^) (Addgene, 185137), AAV-DJ rep-cap (Cell Biolabs, VPK-420-DJ), AAV6 rep-cap (Cell Biolabs, VPK-426) and pHelper (Agilent Technologies, 240071) plasmids were used for production of AAVs. Before use, all plasmids were sequence verified via whole-plasmid sequencing through Plasmidsaurus using Oxford Nanopore Technology with custom analysis and annotation.

### AAV production

Detailed protocols for AAV production and titration are available on protocols.io (10.17504/protocols.io.n2bvjnew5gk5/v1 and 10.17504/protocols.io.e6nvw1n47lmk/v1). AAVs were produced and purified according to published methods^77^, with some minor alterations. In brief, HEK293T cells (American Type Culture Collection (ATCC), CRL-3216; RRID: CVCL_0063) were triple transfected with PEI-MAX (Polysciences, 24765) to deliver the rep-cap or iCAP, pHelper and genome packaging plasmids. Viruses were harvested from cells and media and then purified over 15%, 25%, 40% and 60% iodixanol (OptiPrep; Serumwerk, 1893) step gradients. A Type 70 Ti fixed-angle titanium rotor (Beckman Coulter, 337922) at 58,400 r.p.m. for 1.5 h or a Type 70.1 Ti fixed-angle titanium rotor (Beckman Coulter, 342184) at 61,700 r.p.m. for 1.25 h was used, depending on the scale and number of AAVs to be purified simultaneously. Viruses were concentrated using Amicon Ultra-15 or Amicon Ultra-4 filters with a 100-kD size cutoff (MilliporeSigma, UFC9100 and UFC8100) and formulated in sterile DPBS (Thermo Fisher Scientific, 14190144) with 0.001% Pluronic F-68 (Thermo Fisher Scientific, 24040032). AAVs were titered with quantitative PCR (qPCR) by measuring the number of DNase I-resistant viral genomes, relative to a linearized genome plasmid standard. Before injection, AAVs were diluted in sterile saline. Sequences of qPCR primers for titering are provided in Supplementary Table 3.

ssAAV genomes were used for all experiments, except those shown in the following, where scAAV genomes were used: Fig. 2c and Extended Data Fig. 4a–c; Fig. 2d and Extended Data Fig. 4d,e; Extended Data Fig. 5; Fig. 3f,g and Extended Data Fig. 6; as well as indicated parts of Fig. 2b and Supplementary Data Fig. 1.

### Tissue culture

For AAV production, and for some in vitro experiments, HEK293T cells were used (ATCC, CRL-3216; RRID: CVCL_0063). Cells were grown in DMEM (Thermo Fisher Scientific, 10569010) supplemented with 10% defined FBS (Cytiva, SH30070.03).

For small-scale HEK293T experiments, cells were seeded at optimal confluence (50% for transduction, 90% for transfection) in the morning and transfected or transduced in the afternoon. For transfection, Lipofectamine LTX (Thermo Fisher Scientific, 15338100) was used, with 500 ng of total of DNA and 3 μl of transfection reagent. To avoid saturating SpECTr or fluorescent protein signal, 50 ng of DNA (for Extended Data Fig. 7) or 1,000 double-stranded DNA copies per cell (for Supplementary Data Fig. 2) was used, with pUC19 (New England Biolabs, N3041S; RRID: Addgene_50005) used as filler to ensure efficient transfection. For investigation of transcriptional crosstalk with transfection and transduction in vitro (Extended Data Fig. 7), we transduced cells with a 1 × 10^5^ MOI of AAV-DJ, and cells were collected 5 d later. For in situ restriction enzyme digest of AAV concatemers (Fig. 3c–e and Extended Data Fig. 5), an MOI of 1 × 10^6^ AAV-DJ was used, and cells were collected 3 d later. On the morning of collection, we passaged cells 1:10 onto poly-d-lysine-coated coverslips (Neuvitro, GG-12-1.5h-PDL). Once HEK293T cells had attached, the coverslips were washed three times in DPBS and then fixed. For analysis of fluorescent protein expression, cells were fixed with ice-cold 4% paraformaldehyde (PFA; Electron Microscopy Sciences, 15714-S) in 1× PBS for 15 min at 4 °C and stored in 1× PBS at 4 °C until use. For AAV-Zombie or SpECTr, cells were fixed with ice-cold 3:1 methanol:acetic acid (MAA; Sigma-Aldrich, 322415 and A6283) for 15 min at −20 °C and then stored at −20 °C in 70% ethanol until use.

A detailed protocol for mouse primary cortical and hippocampal neuron culture preparation is available on protocols.io (10.17504/protocols.io.8epv52925v1b/v1). For primary neuron cultures, coverslips (Neuvitro, GG-12-1.5h-pre) were prepared by coating with poly-d-lysine (0.1 mg ml^−1^ overnight; Sigma-Aldrich, P6407), poly-l-ornithine (0.01% overnight; Sigma-Aldrich, P4957) and laminin (0.02 mg ml^−1^ overnight; Thermo Fisher Scientific, 23017015). Primary neurons were prepared by pooling cortices and hippocampi from several embryonic day (E) 16.5 embryos and digesting the tissue in 15 U ml^−1^ papain (Sigma-Aldrich, P3125). The cell suspension was then treated with DNase I, and cells were triturated in HBSS (Thermo Fisher Scientific, 14025092), with 5% horse serum (Thermo Fisher Scientific, 16050130), and then centrifuged through 4% BSA. The cell pellet was resuspended in NeuroCult Neuronal Plating Medium (STEMCELL Technologies, 5713), supplemented with 1:50 NeuroCult SM1 (STEMCELL Technologies, 05711), 0.5 mM GlutaMAX (Thermo Fisher Scientific, 35050061) and 3.7 μg ml^−1^ L-glutamic acid (Sigma-Aldrich, 49449), and plated at a density of 60,000 cells per coverslip. At 5 days in vitro (DIV), half the media was exchanged for BrainPhys Neuronal Media (STEMCELL Technologies, 05790), also supplemented with 1:50 NeuroCult SM1. For transduction, AAV was diluted in the added growth media. The removed plating media were saved and combined 1:1 with complete BrainPhys media. To minimize prolonged transduction due to AAVs in culture media, we used the 1:1 mix of conditioned plating media and BrainPhys media to perform a complete media change at 3 h after transduction, with three washes in pre-warmed BrainPhys between the aspiration of the virus-containing media and addition of fresh conditioned media. Subsequently, the media were half-changed with supplemented BrainPhys media every 3 d. Primary neurons were harvested and fixed as described for HEK293T cells above.

### Animals

Animal husbandry and all procedures involving animals were performed in accordance with the Guide for the Care and Use of Laboratory Animals of the National Institutes of Health and approved by the Institutional Animal Care and Use Committee and by the Office of Laboratory Animal Resources at the California Institute of Technology.

Eight-week old male C57BL/6J (strain no. 000664; RRID: IMSR_JAX:000664), C57BL/6J-background *Prkdc*^*scid/scid*^ (strain no. 001913; RRID: IMSR_JAX:001913) and C57BL/6J-background *Rosa26*^*CAG-LSL-tdTomato*^ (strain no. 007914; RRID: IMSR_JAX:007914) mice were obtained from The Jackson Laboratory. Mice were housed 3–4 per cage, on a 12-h light–dark cycle, and had ad libitum access to food and water. For behavioral experiments and EEG recordings, animals were kept in a reverse light cycle; all behavioral assays and recordings were conducted during the dark cycle, between the hours of Zeitgeber time (ZT) 13 and ZT 17.

For most animal experiments, mice were 8.5–9.5 weeks old at the start of experiments (that is, at injection or at the start of behavioral experiments). However, for EEG experiments, the animals were 8.5–12.5 weeks old at the time of surgery, as they needed to be staggered to accommodate the large cohort size. In this case, care was taken to ensure no systemic assignment to experimental groups based on age at experiment onset.

For primary neuron cultures, timed pregnant C57BL/6N (RRID: MGI:2159965) dams were obtained from Charles River Laboratories.

### Retro-orbital injection

A detailed protocol for systemic AAV administration through retro-orbital injection is available on protocols.io (10.17504/protocols.io.36wgqnw73gk5/v1). AAVs were administered via retro-orbital injection during isoflurane anesthesia (1–3% in 95% O_2_/5% CO_2_, provided by nose cone at 1 L min^−1^), followed by administration of 1–2 drops of 0.5% proparacaine to the corneal surface^77^.

### EEG implantation surgery

A detailed protocol for mouse EEG implantation surgery and EEG data collection is available on protocols.io (10.17504/protocols.io.81wgbzj2ygpk/v1). Mice were anesthetized with isoflurane (5% induction, 1% maintenance) and then subcutaneously injected with ketoprofen (5 mg kg^−1^) and buprenorphine XR (3.25 mg kg^−1^). The animals’ heads were fixed in a stereotaxic frame (David Kopf Instruments), with a heating pad to maintain body temperature. The scalp was then sterilized and subcutaneously injected with 1–2 drops of 0.5% bupivacaine, and a 1.5-cm anterior–posterior incision was made to expose the skull. The skull surface was scored with a scalpel, and the EEG headmount (Pinnacle Technology, 8201) was glued to the surface of the skull using cyanoacrylate adhesive. The anterior edge of the headmount was targeted to be 3.5 mm anterior to bregma. A sterile 23-gauge needle was used to pierce the skull underneath each hole in the headmount. EEG screws were implanted through the headmount and into the craniotomy hole; 0.10-inch screws (Pinnacle Technology, 8209) were used for the anterior holes, and 0.12-inch EEG screws (Pinnacle Technology, 8212) were used for the posterior holes. A small amount of silver epoxy (Pinnacle Technology, 8226) was applied to each screw before fully tightening to ensure electrical connection between the screw and the headmount. Continuity of the contacts was assessed with a multimeter. Adhesive cement (C&B Metabond; Parkell, S398, S371 and S396) was used to secure screws and the headmount in place, followed by dental cement to cover the edges of the headmount. Ibuprofen (30 mg kg^−1^) was provided in drinking water for at least 3 d after surgery. Animals were allowed to recover for at least 1 week before EEG recordings.

### Tissue harvest and processing

Tissue was collected 4 weeks after AAV administration, except for animals used in the *Cacna1a* knockout experiments in which tissue was collected 6 weeks (for motor behavior cohort) or 8 weeks (for EEG cohort) after AAV administration. Animals were euthanized via intraperitoneal injection of 100 mg kg^−1^ euthasol.

Details of tissue harvest protocols for AAV-Zombie or SpECTr experiments in tissue can be found on protocols.io (10.17504/protocols.io.14egn6k7yl5d/v1). In brief, animals were transcardially perfused with 30 ml of ice-cold heparinized 1× PBS, and liver and brain were dissected out. For analysis of fluorescent protein expression, one hemisphere of brain and one lobe of liver were submerged in ice-cold 4% PFA formulated in 1× PBS and fixed overnight at 4 °C. The other hemisphere and another lobe of liver were manually dissected into 1-mm^3^ pieces with regions of interest and flash frozen in optimal cutting temperature (OCT) compound (Scigen, 4586) using a dry ice/ethanol bath. OCT blocks were kept at −70 °C until sectioning.

For measurement of viral genomes from bulk DNA, tissue was processed as above, except that unfixed tissue was used immediately for genomic DNA extraction (DNeasy Blood and Tissue Kit; Qiagen, 69504) rather than frozen.

If animals were not used for AAV-Zombie, SpECTr or bulk DNA extraction, then, after perfusion with PBS, animals were perfused with 30 ml of ice-cold 4% PFA in 1× PBS. Relevant tissues were then extracted and post-fixed overnight in 4% PFA in 1× PBS at 4 °C. For sectioning, brain and liver were cryoprotected through immersion in 30% sucrose in 1× PBS. Once the tissue had sunk, it was flash frozen in OCT compound using a dry ice/ethanol bath and kept at −70 °C until sectioning.

Sections were obtained using a cryostat (Leica Biosystems). Fixed tissue was sectioned at 80 μm, collected in 1× PBS and stored at 4 °C until use. Tissue for AAV-Zombie or SpECTr was sectioned at 20 μm, collected on a clean glass slide (Brain Research Laboratories, 2575-plus), allowed to dry and then stored at −70 °C until use.

Immediately before imaging, gut tissue and DRG were optically cleared by overnight room temperature incubation in RIMS^78,79^ and then mounted in RIMS with an iSpacer (SunJin Lab). Gut tissue was cut longitudinally before incubation in RIMS and mounted with the myenteric plexus up.

### Digital droplet PCR

A detailed protocol for quantification of AAV genomes from total DNA with digital droplet PCR (ddPCR) is available on protocols.io (10.17504/protocols.io.8epv5r84dg1b/v1). To measure viral genomes from bulk cortex and liver DNA, ddPCR was used. First, 1 μg of total DNA was digested overnight with 20 U of SmaI (New England Biolabs, R0141) at 25 °C or with 20 U each of KpnI-HF and SpeI-HF (New England Biolabs, R3142 and R3133) at 37 °C. The digests were diluted 1:10, and 5 μl of each dilution was loaded into a 25-μl PCR reaction (Bio-Rad, 1863024). Then, 23 μl of the PCR reaction was used to generate droplets (Bio-Rad, 1863005) on a QX200 Droplet Generator (Bio-Rad). Forty microliters of droplets was transferred to a PCR plate, which was sealed with a pierceable heat seal (Bio-Rad, 1814040 and 1814000), and the PCR was run according to the manufacturer’s protocol. After PCR, droplets were measured with a QX200 Droplet Reader and analyzed using QX Manager software (Bio-Rad, 12010213). Double-quenched FAM-labeled and HEX-labeled probe assays (Integrated DNA Technologies) were used to detect EGFP sequence and W3SL sequence in the same droplets, and the mean of the two resultant concentrations was used. Sequences of ddPCR primer and probe sets are provided in Supplementary Table 3. SmaI and KpnI-HF/SpeI-HF digests yielded similar results; only SmaI digests are shown.

### IHC

A detailed protocol for IHC on mouse brain slices is available on protocols.io (10.17504/protocols.io.5qpvokmq7l4o/v1). IHC, except against Cacna1a, was performed on free-floating sections. For IHC detection of Cacna1a, sections were first mounted onto slides and subjected to heat-induced epitope retrieval by boiling in 1× citrate buffer, pH 6 (Sigma-Aldrich, C9999) for 10 min in a microwave, followed by thorough washing with 1× PBS.

For IHC, sections were blocked in BlockAid Blocking Solution (Thermo Fisher Scientific, B10710) with 0.1% Triton X-100 (Sigma-Aldrich, 93443). Primary and secondary antibodies were diluted in this blocking buffer. Tissue was incubated with primary antibody overnight at 4 °C and with secondary antibody for 2 h at room temperature. After each antibody incubation step, sections were washed three times for 10 min each in 1× PBS with 0.1% Triton X-100. For Hoechst labeling, sections were incubated for 10 min with 1/10,000 Hoechst 33342 (Thermo Fisher Scientific, H3570) in 1× PBS, followed by three washes in 1× PBS. For segmentation of PCs, sections were Nissl stained with 1/50 NeuroTrace 435/455 (Thermo Fisher Scientific, N21479) in 1× PBS, followed by two 1-h room temperature washes and one overnight wash at 4 °C in 1× PBS with 0.1% Triton X-100. Sections were allowed to dry on slides, and then a coverslip was mounted using Prolong Diamond Antifade Mountant (Thermo Fisher Scientific, P36965).

The following primary antibodies and dilutions were used: rabbit anti-Cacna1a (1:100; Alomone Labs, ACC-001; RRID:AB_2039764), chicken anti-GFP (1:1,000; Aves Labs, 1020; RRID:AB_10000240) and rabbit anti-TagRFP (for detection of mRuby2; 1:1,000; a generous gift from Dawen Cai, University of Michigan; Cancer Tools, 155266; RRID: AB_3107169). Fluorophore-conjugated F(ab′)_2_ fragment secondary antibodies (Jackson ImmunoResearch) were used at a 1:1,000 working concentration.

### AAV-Zombie and SpECTr of cultured cells

A detailed protocol for AAV-Zombie and SpECTr on cultured cells is available on protocols.io (10.17504/protocols.io.36wgqnz53gk5/v3). AAV-Zombie and SpECTr protocols and sequences of Zombie barcodes and their split initiator probes were adapted from Askary et al.^45^. Split initiator probes against endogenous genes and reporter transcripts were designed according to Jang et al.^19^. Sequences of HCR-FISH probes against reporter and endogenous transcripts and against Zombie/SpECTr barcodes are provided in Supplementary Table 3.

For detection of ssAAV and scAAV genomes in cell-free conditions (Supplementary Data Fig. 1), we embedded packaged AAVs (AAV-DJ serotype) in high-concentration Matrigel (Corning, 354262). AAVs were first diluted in ice-cold 1× PBS, and 30 μl of that dilution was added to a pre-chilled tube with 270 μl of high-concentration Matrigel. After mixing by pipetting and brief vortexing, 100 μl of this suspension was spread onto a PDL-coated coverslip, in a 24-well plate on ice. After gelation for 30 min at 37 °C, the samples were incubated for 15 min in ice-cold 1× PBS at 4 °C or in MAA at −20 °C.

For AAV-Zombie and SpECTr of Matrigel-embedded AAV samples and of cultured cells on coverslips, a humidified reaction chamber consisting of a 1-ml pipette tip box filled with pre-warmed RNase-free water was used. Parafilm placed on the wafer of the box served as a surface for the in situ transcription reaction. Coverslips, previously fixed in MAA and stored in 70% ethanol, were first washed twice in 1× PBS. Then, 20 μl of transcription mixture per coverslip was prepared according to the manufacturer’s protocol (Thermo Fisher Scientific, AM1334 and AM1330). For simultaneous T7 and SP6 reactions, the T7 buffer was used with 1 μl of each RNA polymerase. For single polymerase reactions, 2 μl of the polymerase was used. Twenty-microliter droplets were pipetted onto the surface of the parafilm. The coverslips were dipped in UltraPure water (Thermo Fisher Scientific, 10977015), quickly dried by touching their edges to a Kimwipe and then placed cell-side down over the droplets. This reaction was incubated at 37 °C for 3 h.

Once the transcription reaction was finished, the coverslips were placed cell-side up into a clean 24-well plate and fixed for 20 min at 4 °C with ice-cold PFA in 1× PBS. This was followed by two 5-min washes in 1× PBS, followed by two 5-min washes in 5× SSC (Thermo Fisher Scientific, AM9770). Samples were then incubated for 15–30 min in pre-warmed probe hybridization buffer, consisting of 2× SSC, 10% ethylene carbonate (Sigma-Aldrich, E26258) and 10% dextran sulfate (Sigma-Aldrich, 3730) at 37 °C. After this incubation, the coverslips were incubated for 12–16 h at 37 °C in hybridization buffer plus 2 nM of each probe. Probes for Zombie barcodes, reporter transcripts and endogenous transcripts were pooled.

After probe hybridization, samples were washed twice for 30 min in stringent wash buffer (2× SSC, 30% ethylene carbonate) at 37 °C and then three times for 15 min in 5× SSC with 0.1% Tween 20 (Sigma-Aldrich, P1379) and then incubated in HCR amplification buffer (2× SSC, 10% ethylene carbonate) for 20–30 min. HCR hairpins (Molecular Technologies) were heated to 95 °C for 90 s and then cooled to room temperature for 30 min in the dark. For HCR on cultured cells, 30 nM hairpin in amplification buffer was used in a 1-h amplification reaction. The samples were then washed four times in 5× SSC with 0.1% Tween 20 (10 min per wash, at room temperature).

In some cases, the cytoplasm was labeled with a fluorophore-conjugated poly(dT_30_) probe (Integrated DNA Technologies). Coverslips were incubated with 100 nM poly(dT_30_) probe in 5× SSC with 0.1% Tween 20 for 1 h, followed by four 10-min, room temperature washes in 5× SSC with 0.1% Tween 20. Finally, Hoechst 33342 was used to label cell nuclei. Samples were mounted with Prolong Diamond Antifade Mountant.

For co-detection of AAV genomes and capsids, a mouse anti-AAV VP1/VP2/VP3 monoclonal antibody conjugated to Alexa Fluor 488 was used (Clone B1; Progen, 61058-488; RRID: AB_3107170). After poly(dT) labeling, the samples were immunolabeled as described above, with an overnight 4 °C incubation with a 1:100 dilution of the primary antibody in blocking buffer.

For in situ restriction enzyme digest, coverslips were treated with restriction enzymes after MAA fixation and before in situ transcription. Restriction enzyme digests were carried out overnight, at 25 °C for SmaI (New England Biolabs, R0141) and at 37 °C for MscI (New England Biolabs, R0534) and BstEII-HF (New England Biolabs, R3162).

### AAV-Zombie and SpECTr of tissue sections

A detailed protocol for AAV-Zombie and SpECTr on tissue sections is available on protocols.io (10.17504/protocols.io.14egn6k7yl5d/v1). AAV-Zombie and SpECTr were performed on tissue sections as described above for cultured cells, save for a few differences. Incubations in tissue were performed in a staining tray (Simport, M918), and fixation and washes were done in Coplin jars.

Sliced fresh tissue was first removed from −70 °C storage and allowed to warm to room temperature. Slides were then briefly washed with 1× PBS to remove OCT compound and then fixed for 3 h in MAA at −20 °C. Residual fixative was washed off with 1× PBS while the transcription mix was prepared. A total of 200 μl of transcription mix was used per slide, which was pipetted onto the slide and spread out with a clean glass coverslip. We found that simultaneous T7 and SP6 transcription in tissue yielded relatively few and small spots from the SP6-driven barcode. Thus, we carried out T7 and SP6 transcription reactions on separate slides. Likewise, T7 RNA polymerase was used at a 1:10 dilution, whereas SP6 RNA polymerase was used at a 1:5 dilution. As with cultured cells, in situ transcription was carried out at 37 °C for 3 h.

For the HCR-FISH steps on tissue sections, we used 4 nM of each probe in an overnight 37 °C hybridization. The HCR hairpin concentration was also doubled to 60 nM. Short HCR incubations may result in low signal for endogenous transcripts, whereas long incubations can yield large, unresolvable Zombie barcode spots. Thus, we did an overnight incubation with only hairpins for endogenous transcripts and then switched the amplification solution to one containing all hairpins for 1 h.

### Controls for AAV-Zombie and SpECTr

Guidelines for designing, imaging and analyzing AAV-Zombie and SpECTr experiments are available on protocols.io (10.17504/protocols.io.n2bvjn72pgk5/v1). Both AAV-Zombie and SpECTr can produce signals due to hybridization of probes directly to single-stranded AAV genomes and/or transcriptional activity of the AAV ITRs producing barcoded transcripts (for example, faint ‘concatemer’ signal in Genome A condition; Fig. 3b). Thus, controls are necessary for setting thresholds for determining real versus artifactual signal. A non-transduced/non-transfected control sample was used for all AAV-Zombie and SpECTr experiments. For SpECTr experiments, a barcode-only control was used to define signal from probe hybridizing to the AAV genome and/or barcoded transcripts produced due to transcriptional activity of the ITR. As the transcriptional activity of the AAV ITR may differ between cell types, these control experiments were performed in each cell and tissue of interest and processed side by side with experimental samples to mitigate assay-to-assay variability. Depending on the needs of the experiment, other controls may have been included and are outlined in the description of those experiments.

### Imaging

For imaging of fluorescent protein expression in cultured cells and for obtaining whole section images of mouse brain and liver, a Keyence BZ-X710 epifluorescence microscope was used, with a ×10, 0.45 numerical aperture (NA) air objective.

For all other imaging, a Zeiss LSM 880 was used. Imaging of fluorescent protein expression and IHC-stained tissue was accomplished with a ×10, 0.45 NA air objective. Imaging of AAV-Zombie and SpECTr signal in Matrigel, cultured cells and in tissue was performed with a ×63, 1.4 NA oil immersion objective. Imaging settings were chosen to capture full dynamic range of the signal without saturating pixels. When possible, laser power was adjusted before adjusting detector gain. Imaging settings were first optimized on control samples, before imaging of experimental samples. Fields of view were chosen while imaging non-experimental channels (for example, Hoechst or Nissl).

### Image analysis for fluorescent protein expression and IHC

For all cell and nuclear segmentation, except segmentation of PCs, Cellpose^80^ (version 3.0.7; https://www.cellpose.org/; RRID: SCR_021716) was used. Images were batch processed using napari^81^ (version 0.4.19.post1; https://napari.org/stable/; RRID: SCR_022765) and the serialcellpose plugin (version 0.2.2; https://www.napari-hub.org/plugins/napari-serialcellpose). An Anaconda (version 2.5.4; https://www.anaconda.com/; RRID: SCR_025572) distribution of Python (version 3.10.14; https://www.python.org/; RRID: SCR_008394) was used. For HEK293T cells, masks were generated from phase-contrast images. For images of cortex, the fluorescent protein signal was used to generate masks.

PC bodies were segmented manually using the Fiji^82^ distribution of ImageJ (version 1.54f; https://fiji.sc/; RRID: SCR_002285), from images of Nissl-stained tissue (Extended Data Fig. 1d). The large size and intense Nissl staining of the PC body, relative to neighboring cells, was used to identify PCs.

For analysis of fluorescent protein intensity in HEK293T cells, cortical cells and PCs, CellProfiler^83^ (version 4.2.5; https://cellprofiler.org/; RRID: SCR_007358) was used. Classification of cortical cells and PCs as fluorescent protein (XFP) positive or XFP negative was also done using CellProfiler. For PCs, we determined the threshold for using empirically determined thresholds based on negative control tissue. For classification of PCs as mRuby2 positive or mRuby2 negative (Figs. 1d and 4b), a threshold of 25.5 a.u. was used, based on measured intensity of mRuby2 signal in WT animals injected with 3 × 10^11^ vg of only mDLX-minBG-CI-mRuby2 (Fig. 4a,b). For classification of cortical cells as mRuby2 positive or mRuby2 negative (Fig. 4f,h and Extended Data Fig. 3b,c), a threshold of 19.125 a.u. was used, based on measured intensity of segmented cortical cells from the mRuby2 channel for ‘no enhancer’ control animals (Fig. 1f). As Cellpose reliably did not detect GFP cells in the ‘no enhancer’ condition (Fig. 1f), no threshold was necessary for classification of cortical cells as EGFP positive or EGFP negative (Fig. 4f,h). The same threshold was used for all relevant experiments and was measured in animals injected with the lowest dose of the relevant AAV, to provide the most stringent threshold. For these analyses of cortical cells and PCs, three planes (850 μm × 850 μm) from at least four non-adjacent sagittal sections were quantified (that is, at least 12 volumes per animal).

Bulk protein quantification of SCID and WT mice was performed using Fiji, from three non-adjacent 100-μm sections per tissue per animal. Cortex and cerebellum were manually segmented from sagittal sections; liver sections were analyzed whole.

To quantify CRISPR–Cas9 editing of the Ai14 locus, tdTomato-positive cells were manually counted using Fiji. Three volumes (850 μm × 850 μm × 64 μm) were captured from each of at least four non-adjacent sections per animal. PCs and non-PCs were differentiated based on distinct cell morphology and location.

For analysis of Cacna1a expression in cerebellum, Fiji was also used. Four maximum intensity projections of 850 μm × 850 μm × 30 μm volumes were analyzed per animal. In each image, the molecular layer (ML) and the granular layer (GL) were manually segmented, and the total average fluorescence intensity was measured in those regions. For each image, the ML intensity was divided by the GL intensity, and then a per-animal average was determined.

### Image analysis for AAV-Zombie and SpECTr

Guidelines for designing, imaging and analyzing AAV-Zombie and SpECTr experiments are available on protocols.io (10.17504/protocols.io.n2bvjn72pgk5/v1). For analysis of AAV-Zombie and SpECTr spots, segmentation was performed as described above. For primary neurons and HEK293T cells, cell body masks were generated from poly(dT)-TAMRA signal and nuclear masks from Hoechst signal. PC nuclei were manually segmented in Fiji, using large nucleus size, euchromatic nuclear staining and the presence of *Itpr1* transcript to positively identify PCs.

Quantification and measurement of AAV genomes and concatemers in PCs was accomplished using CellProfiler. Genome and concatemer spots were identified within segmented nuclear masks, using empirically determined spot size thresholds and robust background intensity thresholding, chosen due to the sparse foreground signal.

AAV genomes, concatemers and capsid puncta were identified in primary neurons and HEK293T cells as described above, with some exceptions. For both HEK293T cells and primary neurons, masks were size filtered, using empirically determined thresholds. Primary neuron masks were further filtered for presence of an overlapping nuclear mask, and a cytoplasmic mask was generated by subtracting the nuclear mask from the cell body mask. EGFP transcript intensity was measured in the entire cell body mask; AAV genome, concatemer and capsid puncta were quantified in both cytoplasm and nucleus. For HEK293T cells, only nuclear AAV genomes and concatemers were measured.

### Animal behavior

Detailed protocols for the following behavioral assays are available on protocols.io (10.17504/protocols.io.6qpvr8jbzlmk/v2). On each day of behavioral training and data collection, animals were acclimated to the testing room for at least 30 min before measurements were taken. Animals were trained on beam crossing and gait measurement assays 1–2 weeks before experimental measurements started. Behavior equipment was disinfected and deodorized between each animal or, in the case of the open field test, between each cage.

The open field apparatus consisted of four square arenas (27 cm × 27 cm), with a camera (EverFocus, EQ700) placed 1.83 m above the floor of the arenas. An EthoVision XT (Noldus, version 17.5; https://www.noldus.com/ethovision-xt; RRID: SCR_000441) was used to capture and subsequently analyze animal locomotion. Each trial consisted of a 2-min habituation period followed by a 10-min test period. To avoid confounds due to odors from non-cagemates, only animals from the same cage were recorded simultaneously. The average velocity over the course of the experimental period was determined.

The inverted wire hang test was used to measure limb strength^84^. Animals were placed onto a wire mesh screen (6 mm × 6 mm mesh), which was then inverted over the top of a 45-cm-tall cylinder with clean bedding in the bottom. A blinded experimenter recorded the latency to fall within a maximum trial period of 120 s. Three trials were recorded, and the average of those three trials was used.

To measure skilled locomotion using the narrowing beam assay, a clear plexiglass beam consisting of three 25-cm segments (widths 3.5 cm, 2.5 cm and 1.5 cm) was elevated above the table surface using empty clean cages, according to published protocol^85^. At the narrow end, an empty cage was placed on its side, and bedding from the animal’s home cage was placed inside. A white light was also placed over the broad end to motivate animals to move across the beam. For each trial, animals were placed at the end of the widest segment, with all four limbs touching the beam surface. Each trial was recorded with a video camera placed to the side and perpendicular to the beam’s length, affording a view of both left and right hindlimbs. A trial was considered complete once the animal had traversed the beam, without turning around, and entered the goal cage. Once an animal had completed three trials, the session was completed. For each trial, a blinded experimenter measured the animal’s time to cross the beam (ignoring time spent paused), and assigned a neurological score^86^: (7) traverses the beam successfully, with no more than four foot slips and does not grip the side of the beam; (6) traverses the beam successfully, using hindlimbs to aid in more than 50% of strides; (5) traverses the beam successfully, using hindlimbs to aid in less than 50% of strides; (4) traverses the beam successfully, using a hindlimb at least once to push forward but without bearing load on limb; (3) traverses beam successfully, by dragging hindlimbs without using them to push forward; (2) moves at least one body length but fails to traverse beam in the 120-s trial period or falls off; and (1) fails to traverse beam or falls off and does not move more than one body length. The average score and traversal time of the three trials was used for data presentation and statistics.

For gait analysis, we used MouseWalker (https://github.com/MouseWalker/MouseWalker/tree/v1), according to published protocols for hardware design and analysis^87,88^. A clear acrylic platform, 80 cm long, with a 5.3-cm corridor flanked by 12.5-cm-high walls was used. LED lights positioned around the platform enable tracking of animal contacts with the platform surface, through frustrated total internal reflection (fTIR) that is captured using a camera (Apple, iPhone 12 Pro) positioned under the platform. Mice were placed on one end of the corridor, and fTIR was recorded as the animal moved across the platform. Animals were recorded until they had completed three continuously moving traversals of the field of view. Data were analyzed using MouseWalker, and the resulting paw and body tracking was manually inspected by a blinded experimenter to ensure accuracy. In some cases, trials were excluded due to poor tracking.

### EEG recording and analysis

A detailed protocol for mouse EEG implantation surgery and EEG data collection is available on protocols.io (10.17504/protocols.io.81wgbzj2ygpk/v1). EEG recordings were conducted in clear Plexiglas cylinders (25 cm wide, 30 cm high) with ad libitum water. Mice were connected to a pre-amplifier (100× gain, 0.5-Hz high-pass EEG filter; Pinnacle Technology, 8208-SL), which was attached to a commutator (Pinnacle Technology, 8204). Data were acquired by Sirenia Acquisition (Pinnacle Technology, version 2.2.12; https://www.pinnaclet.com/software.html; RRID: SCR_016183), using a Pinnacle data conditioning and acquisition system (Pinnacle Technology, 8206), at a sampling rate of 400 Hz.

Mice were first habituated to the chamber for one session, at least 1 d before recordings began. For each session, a minimum of 90 min was recorded; only the last 60 min were analyzed. To assess ethosuximide blockade of absence seizures, mice were recorded for 90 min and then received a single intraperitoneal injection of ethosuximide (200 mg kg^−1^ in sterile saline; Sigma-Aldrich, E7138) and then were recorded for another 90 min. Only the last 60 min of the pre-ethosuximide and post-ethosuximide recordings were analyzed. Ethosuximide blockade experiments were performed 8 weeks after AAV injection.

EEG signal was analyzed using Sirenia Seizure Pro (Pinnacle Technology, version 2.2.13; https://www.pinnaclet.com/software.html; RRID: SCR_016184). The raw EEG signal was first bandpass filtered (1–25 Hz). A sliding window (0.8 s wide, 0.4-s increments) was used to automatically detect absence seizures using the following criteria: a root mean square (RMS) power exceeding 50 μV in the 5–8-Hz band and a mean amplitude at least two-fold higher than the baseline defined during the pre-injection recording session.

### Statistics and reproducibility

Several biological replicates for each experiment are included in the corresponding figure legends. No data were excluded from analyses, except for gait analysis trials in which paw or body tracking was determined by a blinded experimenter to be inaccurate. For all violin plots, the middle dashed line is the median, and the upper and lower dashed lines are quartiles. Statistical analysis was performed with GraphPad Prism (version 10.0.3, GraphPad Software; RRID: SCR_002798) as described in the figure legends. Where relevant, all tests were two-tailed and corrected for multiple comparisons to maintain an experiment-wide alpha of 0.05.

The following in vivo experiments were repeated once (*n* > 2 animals per experimental condition) with similar results: Fig. 1a,b and Extended Data Fig. 1a; Fig. 2d and Extended Data Fig. 4d,e; Fig. 4a–d and Extended Data Fig. 8a–e; Fig. 5a–c; Fig. 6b and Extended Data Fig. 9a,b; and Supplementary Data Fig. 3a–c. The remaining in vivo experiments were not independently repeated. All in vitro experiments were repeated at least twice with similar results.

### Reporting summary

Further information on research design is available in the Nature Portfolio Reporting Summary linked to this article.

## Online content

Any methods, additional references, Nature Portfolio reporting summaries, source data, extended data, supplementary information, acknowledgements, peer review information; details of author contributions and competing interests; and statements of data and code availability are available at 10.1038/s41587-025-02565-4.

## Supplementary information

Supplementary Information Supplementary Data Figs. 1–3, captions for Supplementary Videos 1 and 2 and Supplementary Table 1

Reporting Summary

Supplementary Video 1 Representative videos of narrowing beam crossing performance for animals in ubiquitous SaCas9 condition. For display purposes, videos are trimmed to show crossing of a 2.5-cm-wide segment of beam. The entire length of the beam was used for data analysis. Videos show the same animals pre-injection and 4 weeks post-injection.

Supplementary Video 2 Representative videos of narrowing beam crossing performance for animals in crosstalk-mediated PC-specific SaCas9 condition. For display purposes, videos are trimmed to show crossing of a 2.5-cm-wide segment of beam. The entire length of the beam was used for data analysis. Videos show the same animals pre-injection and 4 weeks post-injection.

Supplementary Table 2 Supplementary table listing novel plasmids deposited to Addgene (tab 1) and key resources used in the experiments (tab 2).

Supplementary Table 3 Supplementary table listing key DNA sequences used in the experiments, including AAV titering primers (tab 1), ddPCR primer and probe sets (tab 2), FISH probes (tab 3), Zombie FISH probes (tab 4), sgRNA sequences (tab 5) and other sequence elements, such as promoters and enhancers (tab 6).

## Data Availability

All sequences of primers, probes, sgRNAs and other sequence elements are provided in Supplementary Table 3. Images of brain tissue that are quantified in this work are deposited in the Brain Image Library (10.35077/g.1163). Tabular datasets and behavior videos supporting the conclusions of this work are available on Zenodo (10.5281/zenodo.13952929)^89^. All other data that support the findings of this study are available from the corresponding authors upon reasonable request.

## References

[1] Challis, R. C. et al. Adeno-associated virus toolkit to target diverse brain cells. *Annu. Rev. Neurosci.* **45**, 447–469 (2022). 35440143 10.1146/annurev-neuro-111020-100834

[2] Wang, D., Tai, P. W. L. & Gao, G. Adeno-associated virus vector as a platform for gene therapy delivery. *Nat. Rev. Drug Discov.* **18**, 358–378 (2019). 30710128 10.1038/s41573-019-0012-9 PMC6927556

[3] Zhu, D., Schieferecke, A. J., Lopez, P. A. & Schaffer, D. V. Adeno-associated virus vector for central nervous system gene therapy. *Trends Mol. Med.* **27**, 524–537 (2021). 33895085 10.1016/j.molmed.2021.03.010

[4] Nectow, A. R. & Nestler, E. J. Viral tools for neuroscience. *Nat. Rev. Neurosci.* **21**, 669–681 (2020). 33110222 10.1038/s41583-020-00382-z PMC7808553

[5] Campos, L. J. et al. Advances in AAV technology for delivering genetically encoded cargo to the nonhuman primate nervous system. *Curr. Res. Neurobiol.* **4**, 100086 (2023). 37397806 10.1016/j.crneur.2023.100086 PMC10313870

[6] Berns, K. I. & Muzyczka, N. AAV: an overview of unanswered questions. *Hum. Gene Ther.* **28**, 308–313 (2017). 28335618 10.1089/hum.2017.048 PMC5399733

[7] Penaud-Budloo, M. et al. Adeno-associated virus vector genomes persist as episomal chromatin in primate muscle. *J. Virol.* **82**, 7875–7885 (2008). 18524821 10.1128/JVI.00649-08 PMC2519600

[8] Duan, D. et al. Circular intermediates of recombinant adeno-associated virus have defined structural characteristics responsible for long-term episomal persistence in muscle tissue. *J. Virol.* **72**, 8568–8577 (1998). 9765395 10.1128/jvi.72.11.8568-8577.1998 PMC110267

[9] Nakai, H. et al. Extrachromosomal recombinant adeno-associated virus vector genomes are primarily responsible for stable liver transduction in vivo. *J. Virol.* **75**, 6969–6976 (2001). 11435577 10.1128/JVI.75.15.6969-6976.2001 PMC114425

[10] Schnepp, B. C. et al. Recombinant adeno-associated virus vector genomes take the form of long-lived, transcriptionally competent episomes in human muscle. *Hum. Gene Ther.* **27**, 32–42 (2016). 26650966 10.1089/hum.2015.136 PMC5374867

[11] Yang, J. et al. Concatamerization of adeno-associated virus circular genomes occurs through intermolecular recombination. *J. Virol.* **73**, 9468–9477 (1999). 10516055 10.1128/jvi.73.11.9468-9477.1999 PMC112981

[12] Deverman, B. E. et al. Cre-dependent selection yields AAV variants for widespread gene transfer to the adult brain. *Nat. Biotechnol.* **34**, 204–209 (2016). 26829320 10.1038/nbt.3440 PMC5088052

[13] Chan, K. Y. et al. Engineered AAVs for efficient noninvasive gene delivery to the central and peripheral nervous systems. *Nat. Neurosci.* **20**, 1172–1179 (2017). 28671695 10.1038/nn.4593 PMC5529245

[14] Ravindra Kumar, S. et al. Multiplexed Cre-dependent selection yields systemic AAVs for targeting distinct brain cell types. *Nat. Methods* **17**, 541–550 (2020). 32313222 10.1038/s41592-020-0799-7 PMC7219404

[15] Goertsen, D. et al. AAV capsid variants with brain-wide transgene expression and decreased liver targeting after intravenous delivery in mouse and marmoset. *Nat. Neurosci.* **25**, 106–115 (2022). 34887588 10.1038/s41593-021-00969-4

[16] Chen, X. et al. Engineered AAVs for non-invasive gene delivery to rodent and non-human primate nervous systems. *Neuron* **110**, 2242–2257 (2022). 35643078 10.1016/j.neuron.2022.05.003 PMC9308721

[17] Chen, X. et al. Functional gene delivery to and across brain vasculature of systemic AAVs with endothelial-specific tropism in rodents and broad tropism in primates. *Nat. Commun.* **14**, 3345 (2023). 37291094 10.1038/s41467-023-38582-7 PMC10250345

[18] Chuapoco, M. R. et al. Adeno-associated viral vectors for functional intravenous gene transfer throughout the non-human primate brain. *Nat. Nanotechnol.* **18**, 1241–1251 (2023). 37430038 10.1038/s41565-023-01419-x PMC10575780

[19] Jang, M. J. et al. Spatial transcriptomics for profiling the tropism of viral vectors in tissues. *Nat. Biotechnol.* **41**, 1272–1286 (2023). 36702899 10.1038/s41587-022-01648-w PMC10443732

[20] Tabebordbar, M. et al. Directed evolution of a family of AAV capsid variants enabling potent muscle-directed gene delivery across species. *Cell* **184**, 4919–4938 (2021). 34506722 10.1016/j.cell.2021.08.028 PMC9344975

[21] Weinmann, J. et al. Identification of a myotropic AAV by massively parallel in vivo evaluation of barcoded capsid variants. *Nat. Commun.* **11**, 5432 (2020). 33116134 10.1038/s41467-020-19230-w PMC7595228

[22] Nonnenmacher, M. et al. Rapid evolution of blood-brain-barrier-penetrating AAV capsids by RNA-driven biopanning. *Mol. Ther. Methods Clin. Dev.* **20**, 366–378 (2021). 33553485 10.1016/j.omtm.2020.12.006 PMC7841218

[23] Hanlon, K. S. et al. Selection of an efficient AAV vector for robust CNS transgene expression. *Mol. Ther. Methods Clin. Dev.* **15**, 320–332 (2019). 31788496 10.1016/j.omtm.2019.10.007 PMC6881693

[24] Stanton, A. C. et al. Systemic administration of novel engineered AAV capsids facilitates enhanced transgene expression in the macaque CNS. *Med* **4**, 31–50 (2023). 36417917 10.1016/j.medj.2022.11.002 PMC9840684

[25] Dimidschstein, J. et al. A viral strategy for targeting and manipulating interneurons across vertebrate species. *Nat. Neurosci.* **19**, 1743–1749 (2016). 27798629 10.1038/nn.4430 PMC5348112

[26] Hrvatin, S. et al. A scalable platform for the development of cell-type-specific viral drivers. *eLife* **8**, e48089 (2019). 31545165 10.7554/eLife.48089 PMC6776442

[27] Graybuck, L. T. et al. Enhancer viruses for combinatorial cell-subclass-specific labeling. *Neuron* **109**, 1449–1464 (2021). 33789083 10.1016/j.neuron.2021.03.011 PMC8610077

[28] Mich, J. K. et al. Functional enhancer elements drive subclass-selective expression from mouse to primate neocortex. *Cell Rep.* **34**, 108754 (2021). 33789096 10.1016/j.celrep.2021.108754 PMC8163032

[29] Mich, J. K. et al. Enhancer-AAVs allow genetic access to oligodendrocytes and diverse populations of astrocytes across species. Preprint at *bioRxiv*10.1101/2023.09.20.558718 (2023).

[30] Lawler, A. J. et al. Machine learning sequence prioritization for cell type-specific enhancer design. *eLife* **11**, e69571 (2022). 35576146 10.7554/eLife.69571 PMC9110026

[31] Fornes, O. et al. OnTarget: in silico design of MiniPromoters for targeted delivery of expression. *Nucleic Acids Res.* **51**, W379–W386 (2023). 37166953 10.1093/nar/gkad375 PMC10320062

[32] Lambert, J. T. et al. Parallel functional testing identifies enhancers active in early postnatal mouse brain. *eLife* **10**, e69479 (2021). 34605404 10.7554/eLife.69479 PMC8577842

[33] Rubin, A. N. et al. Regulatory elements inserted into AAVs confer preferential activity in cortical interneurons. *eNeuro* **7**, ENEURO.0211-20.2020 (2020). 10.1523/ENEURO.0211-20.2020 PMC7768279 33199411

[34] Vormstein-Schneider, D. et al. Viral manipulation of functionally distinct interneurons in mice, non-human primates and humans. *Nat. Neurosci.* **23**, 1629–1636 (2020). 32807948 10.1038/s41593-020-0692-9 PMC8015416

[35] Kussick, E. et al. Enhancer AAVs for targeting spinal motor neurons and descending motor pathways in rodents and macaque. Preprint at *bioRxiv*10.1101/2024.07.30.605864 (2024). 10.1016/j.celrep.2025.115730 40403722

[36] Doudna, J. A. The promise and challenge of therapeutic genome editing. *Nature* **578**, 229–236 (2020). 32051598 10.1038/s41586-020-1978-5 PMC8992613

[37] Porteus, M. H. A new class of medicines through DNA editing. *N. Engl. J. Med.* **380**, 947–959 (2019). 30855744 10.1056/NEJMra1800729

[38] Zhang, F. Development of CRISPR–Cas systems for genome editing and beyond. *Q. Rev. Biophys.* **52**, e6 (2019).

[39] Field, A. & Adelman, K. Evaluating enhancer function and transcription. *Annu. Rev. Biochem.* **89**, 213–234 (2020). 32197056 10.1146/annurev-biochem-011420-095916

[40] Panigrahi, A. & O’Malley, B. W. Mechanisms of enhancer action: the known and the unknown. *Genome Biol.* **22**, 108 (2021). 33858480 10.1186/s13059-021-02322-1 PMC8051032

[41] Duan, D., Yue, Y., Yan, Z. & Engelhardt, J. F. A new dual-vector approach to enhance recombinant adeno-associated virus-mediated gene expression through intermolecular *cis* activation. *Nat. Med.* **6**, 595–598 (2000). 10802719 10.1038/75080

[42] Williams, R. W. & Herrup, K. The control of neuron number. *Annu. Rev. Neurosci.* **11**, 423–453 (1988). 3284447 10.1146/annurev.ne.11.030188.002231

[43] Wang, S. K., Lapan, S. W., Hong, C. M., Krause, T. B. & Cepko, C. L. In situ detection of adeno-associated viral vector genomes with SABER-FISH. *Mol. Ther. Methods Clin. Dev.* **19**, 376–386 (2020). 33209963 10.1016/j.omtm.2020.10.003 PMC7658570

[44] Zhao, J. et al. High-resolution histological landscape of AAV DNA distribution in cellular compartments and tissues following local and systemic injection. *Mol. Ther. Methods Clin. Dev.* **18**, 856–868 (2020). 32953935 10.1016/j.omtm.2020.08.006 PMC7479330

[45] Askary, A. et al. In situ readout of DNA barcodes and single base edits facilitated by in vitro transcription. *Nat. Biotechnol.* **38**, 66–75 (2020). 31740838 10.1038/s41587-019-0299-4 PMC6954335

[46] Choi, H. M. T. et al. Third-generation in situ hybridization chain reaction: multiplexed, quantitative, sensitive, versatile, robust. *Development* **145**, dev165753 (2018). 10.1242/dev.165753 PMC6031405 29945988

[47] De Leeuw, C. N. et al. rAAV-compatible MiniPromoters for restricted expression in the brain and eye. *Mol. Brain* **9**, 52 (2016). 27164903 10.1186/s13041-016-0232-4 PMC4862195

[48] Juven-Gershon, T., Cheng, S. & Kadonaga, J. T. Rational design of a super core promoter that enhances gene expression. *Nat. Methods* **3**, 917–922 (2006). 17124735 10.1038/nmeth937

[49] Boshart, M. et al. A very strong enhancer is located upstream of an immediate early gene of human cytomegalovirus. *Cell* **41**, 521–530 (1985). 2985280 10.1016/s0092-8674(85)80025-8

[50] Duan, D., Yue, Y. & Engelhardt, J. F. Consequences of DNA-dependent protein kinase catalytic subunit deficiency on recombinant adeno-associated virus genome circularization and heterodimerization in muscle tissue. *J. Virol.* **77**, 4751–4759 (2003). 12663782 10.1128/JVI.77.8.4751-4759.2003 PMC152118

[51] Song, S., Laipis, P. J., Berns, K. I. & Flotte, T. R. Effect Of DNA-dependent protein kinase on the molecular fate of the rAAV2 genome in skeletal muscle. *Proc. Natl Acad. Sci. USA* **98**, 4084–4088 (2001). 11274433 10.1073/pnas.061014598 PMC31183

[52] Nakai, H., Fuess, S., Storm, T. A., Meuse, L. A. & Kay, M. A. Free DNA ends are essential for concatemerization of synthetic double-stranded adeno-associated virus vector genomes transfected into mouse hepatocytes in vivo. *Mol. Ther.* **7**, 112–121 (2003). 12573624 10.1016/s1525-0016(02)00034-5

[53] Nakai, H., Storm, T. A., Fuess, S. & Kay, M. A. Pathways of removal of free DNA vector ends in normal and DNA-PKcs–deficient SCID mouse hepatocytes transduced with rAAV vectors. *Hum. Gene Ther.* **14**, 871–881 (2003). 12828858 10.1089/104303403765701169

[54] Choi, Y.-K., Nash, K., Byrne, B. J., Muzyczka, N. & Song, S. The effect of DNA-dependent protein kinase on adeno-associated virus replication. *PLoS ONE* **5**, e15073 (2010). 21188139 10.1371/journal.pone.0015073 PMC3004791

[55] Choi, V. W., McCarty, D. M. & Samulski, R. J. Host cell DNA repair pathways in adeno-associated viral genome processing. *J. Virol.* **80**, 10346–10356 (2006). 17041215 10.1128/JVI.00841-06 PMC1641795

[56] Maurer, A. C. & Weitzman, M. D. Adeno-associated virus genome interactions important for vector production and transduction. *Hum. Gene Ther.* **31**, 499–511 (2020). 32303138 10.1089/hum.2020.069 PMC7232694

[57] Maeda, N., Niinobe, M. & Mikoshiba, K. A cerebellar Purkinje cell marker P400 protein is an inositol 1,4,5-trisphosphate (InsP3) receptor protein. Purification and characterization of InsP3 receptor complex. *EMBO J.* **9**, 61–67 (1990). 2153079 10.1002/j.1460-2075.1990.tb08080.x PMC551630

[58] Tabebordbar, M. et al. In vivo gene editing in dystrophic mouse muscle and muscle stem cells. *Science* **351**, 407–411 (2016). 26721686 10.1126/science.aad5177 PMC4924477

[59] Stahl, E. C. et al. Genome editing in the mouse brain with minimally immunogenic Cas9 RNPs. *Mol. Ther.* **31**, 2422–2438 (2023). 37403358 10.1016/j.ymthe.2023.06.019 PMC10422012

[60] Monteys, A. M. et al. Regulated control of gene therapies by drug-induced splicing. *Nature* **596**, 291–295 (2021). 34321659 10.1038/s41586-021-03770-2 PMC8966400

[61] Lang, J. F., Toulmin, S. A., Brida, K. L., Eisenlohr, L. C. & Davidson, B. L. Standard screening methods underreport AAV-mediated transduction and gene editing. *Nat. Commun.* **10**, 3415 (2019). 31363095 10.1038/s41467-019-11321-7 PMC6667494

[62] Jun, K. et al. Ablation of P/Q-type Ca^2+^ channel currents, altered synaptic transmission, and progressive ataxia in mice lacking the α_1A_-subunit. *Proc. Natl Acad. Sci. USA* **96**, 15245–15250 (1999). 10611370 10.1073/pnas.96.26.15245 PMC24805

[63] Fletcher, C. F. et al. Dystonia and cerebellar atrophy in *Cacna1a* null mice lacking P/Q calcium channel activity. *FASEB J.* **15**, 1288–1290 (2001). 11344116 10.1096/fj.00-0562fje

[64] Mallmann, R. T. et al. Ablation of Ca_V_2.1 voltage-gated Ca^2+^ channels in mouse forebrain generates multiple cognitive impairments. *PLoS ONE* **8**, e78598 (2013). 24205277 10.1371/journal.pone.0078598 PMC3814415

[65] Rossignol, E., Kruglikov, I., Van Den Maagdenberg, A. M. J. M., Rudy, B. & Fishell, G. Ca_V_2.1 ablation in cortical interneurons selectively impairs fast‐spiking basket cells and causes generalized seizures. *Ann. Neurol.* **74**, 209–222 (2013). 23595603 10.1002/ana.23913 PMC3849346

[66] Bomben, V. C. et al. Isolated P/Q calcium channel deletion in layer VI corticothalamic neurons generates absence epilepsy. *J. Neurosci.* **36**, 405–418 (2016). 26758833 10.1523/JNEUROSCI.2555-15.2016 PMC4710767

[67] Todorov, B. et al. Purkinje cell-specific ablation of Ca_V_2.1 channels is sufficient to cause cerebellar ataxia in mice. *Cerebellum* **11**, 246–258 (2012). 21870131 10.1007/s12311-011-0302-1 PMC3311848

[68] Mark, M. D. et al. Delayed postnatal loss of P/Q-type calcium channels recapitulates the absence epilepsy, dyskinesia, and ataxia phenotypes of genomic *Cacna1A* mutations. *J. Neurosci.* **31**, 4311–4326 (2011). 21411672 10.1523/JNEUROSCI.5342-10.2011 PMC3065835

[69] Zhang, X. et al. Highly restricted expression of Cre recombinase in cerebellar Purkinje cells. *Genesis* **40**, 45–51 (2004). 15354293 10.1002/gene.20062

[70] Ning, K. et al. Inhibition of DNA-dependent protein kinase catalytic subunit boosts rAAV transduction of polarized human airway epithelium. *Mol. Ther. Methods Clin. Dev.* **31**, 101115 (2023). 37841417 10.1016/j.omtm.2023.101115 PMC10568418

[71] Yao, Y. et al. Variants of the adeno-associated virus serotype 9 with enhanced penetration of the blood–brain barrier in rodents and primates. *Nat. Biomed. Eng.* **6**, 1257–1271 (2022). 36217021 10.1038/s41551-022-00938-7

[72] Xu, X. et al. Engineered miniature CRISPR–Cas system for mammalian genome regulation and editing. *Mol. Cell* **81**, 4333–4345 (2021). 10.1016/j.molcel.2021.08.008 34480847

[73] Davis, J. R. et al. Efficient in vivo base editing via single adeno-associated viruses with size-optimized genomes encoding compact adenine base editors. *Nat. Biomed. Eng.* **6**, 1272–1283 (2022). 35902773 10.1038/s41551-022-00911-4 PMC9652153

[74] Davis, J. R. et al. Efficient prime editing in mouse brain, liver and heart with dual AAVs. *Nat. Biotechnol.* **42**, 253–264 (2023). 37142705 10.1038/s41587-023-01758-z PMC10869272

[75] Zhuo, C. et al. Spatiotemporal control of CRISPR/Cas9 gene editing. *Sig. Transduct. Target. Ther.* **6**, 238 (2021). 10.1038/s41392-021-00645-w PMC8214627 34148061

[76] Tsuchida, C. A., Wasko, K. M., Hamilton, J. R. & Doudna, J. A. Targeted nonviral delivery of genome editors in vivo. *Proc. Natl Acad. Sci. USA* **121**, e2307796121 (2024). 38437567 10.1073/pnas.2307796121 PMC10945750

[77] Challis, R. C. et al. Systemic AAV vectors for widespread and targeted gene delivery in rodents. *Nat. Protoc.* **14**, 379–414 (2019). 30626963 10.1038/s41596-018-0097-3 PMC13333184

[78] Yang, B. et al. Single-cell phenotyping within transparent intact tissue through whole-body clearing. *Cell* **158**, 945–958 (2014). 25088144 10.1016/j.cell.2014.07.017 PMC4153367

[79] Treweek, J. B. et al. Whole-body tissue stabilization and selective extractions via tissue-hydrogel hybrids for high-resolution intact circuit mapping and phenotyping. *Nat. Protoc.* **10**, 1860–1896 (2015). 26492141 10.1038/nprot.2015.122 PMC4917295

[80] Stringer, C., Wang, T., Michaelos, M. & Pachitariu, M. Cellpose: a generalist algorithm for cellular segmentation. *Nat. Methods* **18**, 100–106 (2021). 33318659 10.1038/s41592-020-01018-x

[81] Ahlers, J. et al. napari: a multi-dimensional image viewer for Python. *Zenodo*10.5281/ZENODO.3555620 (2023).

[82] Schindelin, J. et al. Fiji: an open-source platform for biological-image analysis. *Nat. Methods* **9**, 676–682 (2012). 22743772 10.1038/nmeth.2019 PMC3855844

[83] Stirling, D. R. et al. CellProfiler 4: improvements in speed, utility and usability. *BMC Bioinform.* **22**, 433 (2021). 10.1186/s12859-021-04344-9 PMC8431850 34507520

[84] Maejima, T. et al. Postnatal loss of P/Q-type channels confined to rhombic-lip-derived neurons alters synaptic transmission at the parallel fiber to Purkinje cell synapse and replicates genomic *Cacna1a* mutation phenotype of ataxia and seizures in mice. *J. Neurosci.* **33**, 5162–5174 (2013). 23516282 10.1523/JNEUROSCI.5442-12.2013 PMC3641643

[85] Fleming, S. M., Ekhator, O. R. & Ghisays, V. Assessment of sensorimotor function in mouse models of Parkinson’s disease. *J. Vis. Exp.* 50303 (2013). 10.3791/50303 PMC3727502 23851663

[86] Carter, R. J., Morton, J. & Dunnett, S. B. Motor coordination and balance in rodents. *Curr. Protoc. Neurosci*. 10.1002/0471142301.ns0812s15 (2001). 10.1002/0471142301.ns0812s15 18428540

[87] Mendes, C. S. et al. Quantification of gait parameters in freely walking rodents. *BMC Biol.* **13**, 50 (2015). 26197889 10.1186/s12915-015-0154-0 PMC4511453

[88] Kiven, S. et al. Spatiotemporal alterations in gait in humanized transgenic sickle mice. *Front. Immunol.* **11**, 561947 (2020). 33178189 10.3389/fimmu.2020.561947 PMC7593487

[89] Coughlin, G. Coughlin_Borsos_et_al. *Zenodo*10.5281/zenodo.13952929 (2024).

