# Supplementary material for: Spatial genomics of AAV vectors reveals mechanism of transcriptional crosstalk that enables targeted delivery of large genetic cargo
Source: Nat Biotechnol. 2025 Mar 20;44(1):133–45. doi: 10.1038/s41587-025-02565-4 (PMC12807873; doi:10.1038/s41587-025-02565-4)
Supplement: Supplementary file 1 — Supplementary Data Figs. 1–3, captions for Supplementary Videos 1 and 2 and Supplementary Table 1 [file 41587_2025_2565_MOESM1_ESM.pdf]

nature biotechnology

Supplementary information https://doi.org/10.1038/s41587-025-02565-4

# Spatial genomics of AAV vectors reveals mechanism of transcriptional crosstalk that enables targeted delivery of large genetic cargo

In the format provided by the authors and unedited

Supplementary information

**Table of contents**

Supplementary Data Figure 1. Methanol and acetic acid fixation is sufficient to denature the AAV capsid, enabling genome detection by AAV-Zombie.

Supplementary Data Figure 2. Relationship between number of T7-barcode repeats and spot area.

Supplementary Data Figure 3. AAV transduction of wildtype and SCID mice.

Caption for Supplementary Video 1. Representative videos of narrowing beam crossing performance for animals in ubiquitous SaCas9 condition.

Caption for Supplementary Video 2. Representative videos of narrowing beam crossing performance for animals in crosstalk-mediated PC-specific SaCas9 condition.

Supplementary Table 1. Indications where transcriptional crosstalk may be therapeutically beneficial for high or cell type-specific expression.

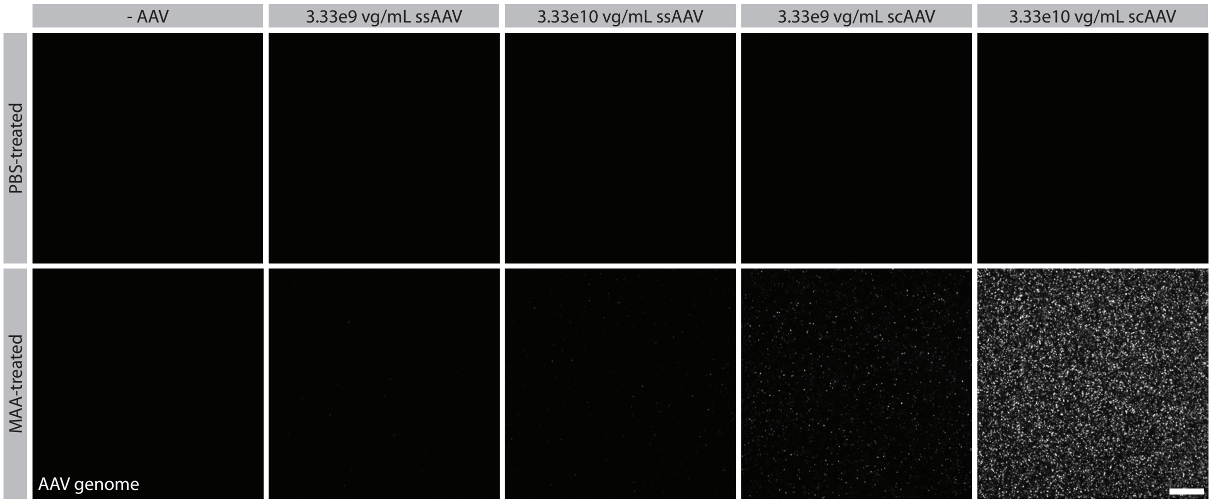


**Supplementary Data Figure 1. Methanol and acetic acid fixation is sufficient to denature the AAV capsid, enabling genome detection by AAV-Zombie.** To investigate whether processing of the AAV genome by the host cell was necessary for detection by AAV-Zombie, we devised a cell-free system. Single-stranded and self-complementary genomes were packaged into AAV-DJ, and then embedded in high-concentration Matrigel. Following gelation, the samples were treated with ice-cold 1x PBS (top row) or MAA (bottom row). MAA treatment resulted in an increase in Zombie signal from scAAV samples, as compared to PBS controls, suggesting that MAA treatment can denature the capsid, releasing the AAV genome. Scale bar = 20 μm.

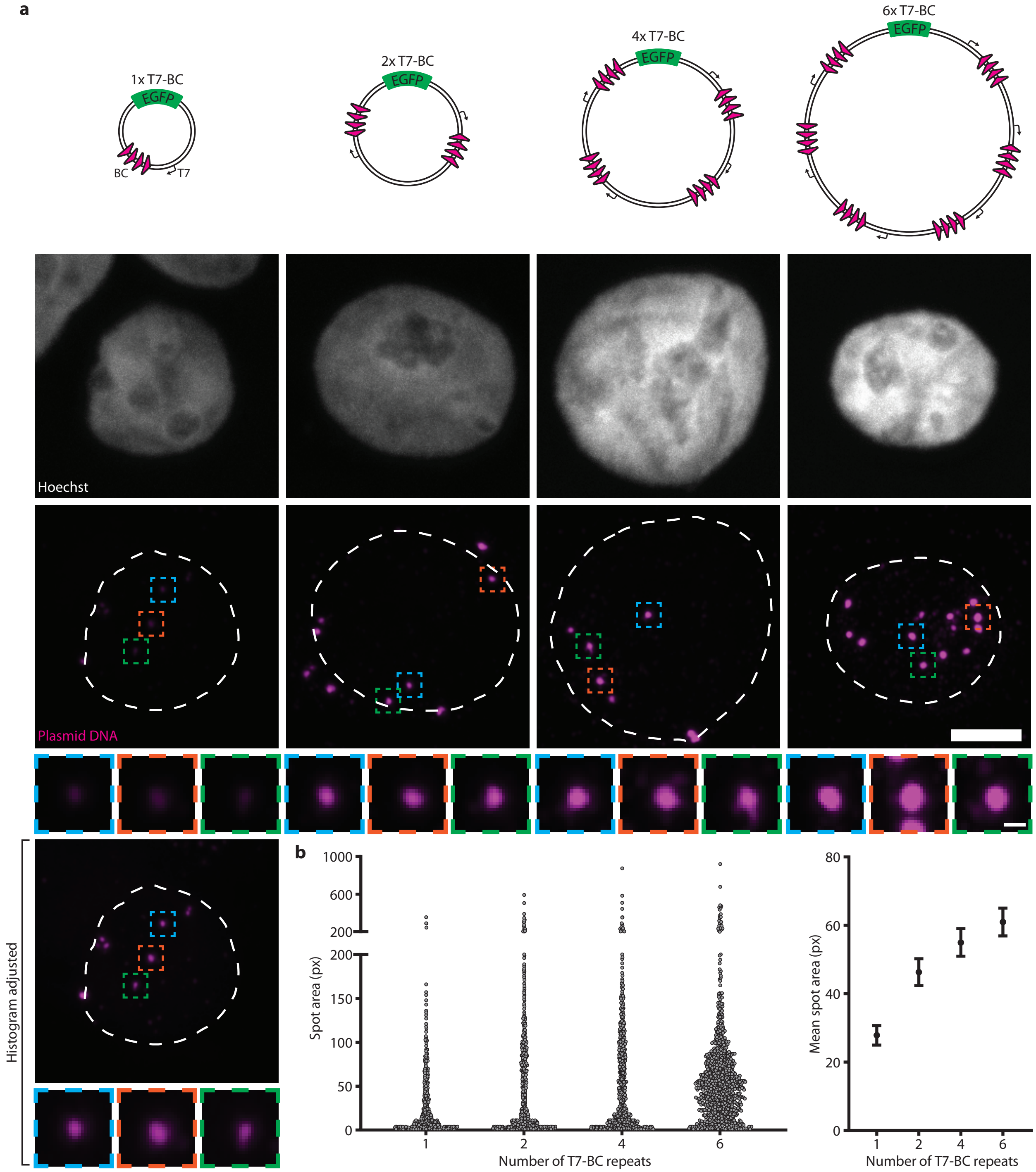


**Supplementary Data Figure 2. Relationship between number of T7-barcode repeats and spot area.** **a,** Plasmids with increasing numbers of T7-barcode (T7-BC) repeats were constructed and transfected at equimolar amounts into HEK293T cells. Zombie was then used to detect the individual plasmids. Scale bar = 20 µm for larger images, 2 µm for insets. **b,** Quantification of spot area as a function of number of

T7-BC repeats. Plasmids with more T7-BC repeats yielded larger spots. $n$ = 706 (1 T7-BC repeat), 932 (2 T7-BC repeats), 1052 (4 T7-BC repeats), 999 (6 T7-BC repeats) spots per condition. Points represent mean and bars are 95% confidence interval.

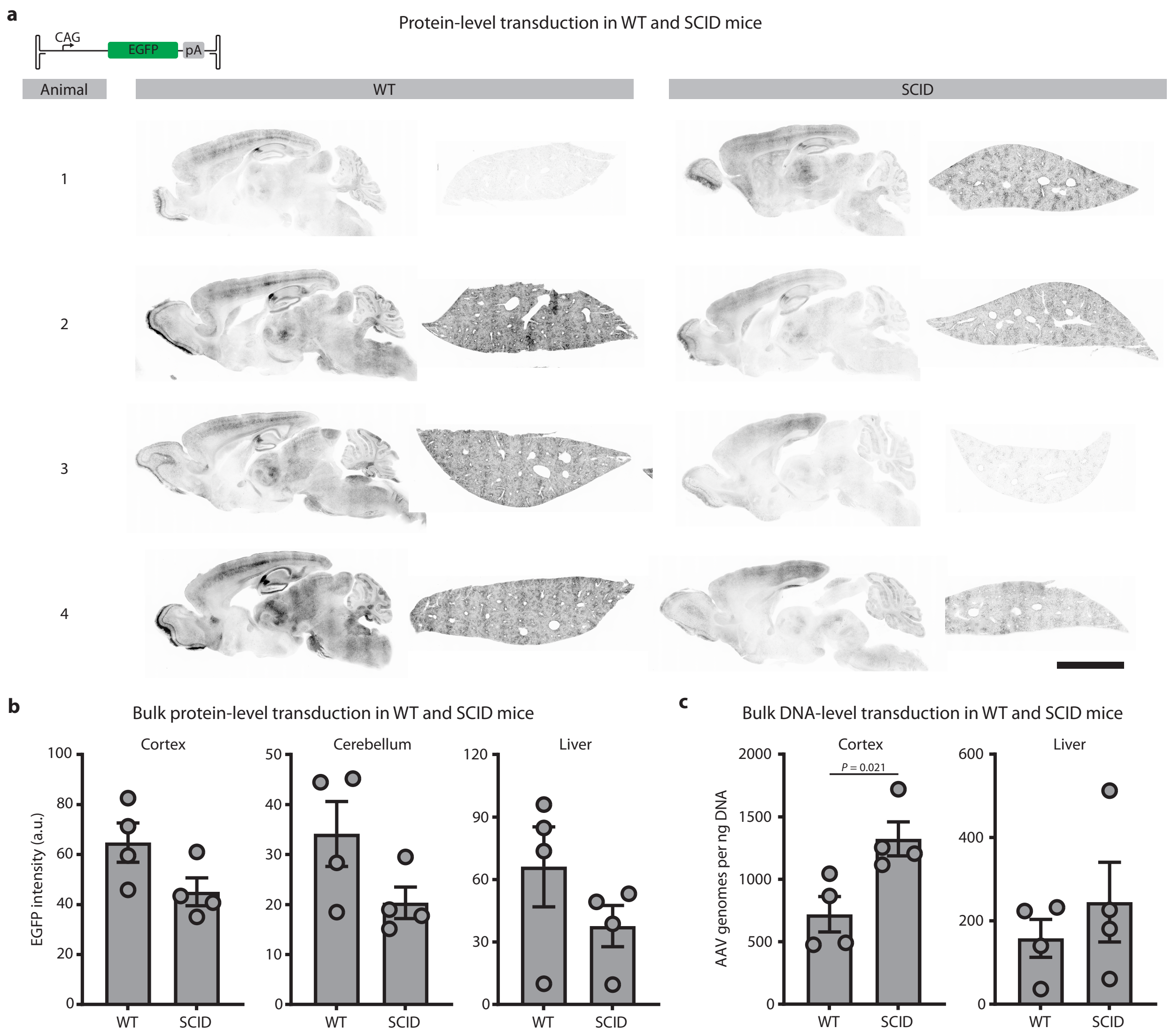


**Supplementary Data Figure 3. AAV transduction of wildtype and SCID mice. a**, Wildtype and SCID C57BL/6J animals were transduced with 3e11 vg of AAV-PHP.eB packaging a CAG-EGFP reporter, and tissue was collected 4 weeks later. Representative sagittal brain (left) and liver (right) sections are shown. Scale bar = 5 mm. **b**, Quantification of bulk protein in cortex (left), cerebellum (middle), and liver (right) of WT and SCID animals, assessed by mean EGFP intensity. **c**, Quantification of bulk viral DNA in WT and SCID cortex and liver, assayed through digital droplet PCR. SmaI digests and KpnI-HF/SpeI-HF digests yielded similar results; results from SmaI-digested samples are shown. For (**b**) and (**c**), statistical significance was determined using unpaired t-tests. $n = 4$ animals per genotype. Bars are mean ± s.e.m.

**Supplementary Video 1. Representative videos of narrowing beam crossing performance for animals in ubiquitous SaCas9 condition.** For display purposes, videos are trimmed to show crossing of 2.5 cm wide segment of beam. The entire length of beam was used for data analysis. Videos show the same animals pre-injection and 4 weeks post-injection.

**Supplementary Video 2. Representative videos of narrowing beam crossing performance for animals in crosstalk-mediated PC-specific SaCas9 condition.** For display purposes, videos are trimmed to show crossing of 2.5 cm wide segment of beam. The entire length of beam was used for data analysis. Videos show the same animals pre-injection and 4 weeks post-injection.

**Supplementary Table 1. Indications where transcriptional crosstalk may be therapeutically beneficial for high or cell type-specific expression.**

| Disorder | Human Gene (bp) | Target Cell Type | Targeting Rationale | References |
|---|---|---|---|---|
| Alpha-1 antitrypsin deficiency | SERPINA1 (1254) | Lung, Liver* | Mutations in SERPINA1 cause accumulation in the liver and underexpression in the lung. Enhancing lung expression while minimizing liver expression would be beneficial. Though this transcript is relatively short, crosstalk-mediated enhancement may be necessary due to potential need for several regulatory motifs on the genome. | 1,2 |
| CDKL5 Deficiency Disorder | CDKL5 (3427) | Cerebral Cortex, Cerebellum | CDKL5 is primarily expressed in neurons, both excitatory and inhibitory, and highly expressed in early development. Inclusion of a temporally sensitive enhancer element could promote high expression during the critical period, while maintaining a lower baseline expression level. | 3 |
| Cornelia De Lange Syndrome | BRD4 (4089), SMC1A (3969) | Cerebral Cortex Neurons, Epithelial Cells, Smooth Muscle and Enteric Nervous System | Brd4 overexpression is linked to cancer progression and inflammatory dysregulation, emphasizing the incentive to restrict unnecessary expression in off-target cell types. Smc1a overexpression can dysregulate chromosomal segregation in mitosis and meiosis. | 4–7 |
| Cystic Fibrosis | CFTR (4440) | Secretory Epithelia in Lung, Pancreas | The coding sequence length for CFTR limits promoter and regulatory element choices in a single AAV genome. Additionally, CFTR is not expressed in the muscle, heart, neurons or blood cells. Expressing chloride channels in non-target cell types could perturb homeostatic processes | 8 |
| Duchenne Muscular Dystrophy | mini- or micro-dystrophin (varies) | Skeletal Muscle | Dystrophin, including miniaturized constructs, have long sequences, which limit the inclusion of gene regulatory elements. | 9 |
| Osteogenesis Imperfecta; Ehlers Danlos Syndrome | COL1A1 (4392) | FSP+ mesenchymal cells, fibroblasts | Subpopulations of COL1A1 producing cells are more specifically responsible for the OI phenotype. Aberrant overexpression of COL1A1 may lead to extensive tissue fibrosis and complications, particularly in the vascular system. | 10,11 |
| Pyruvate Carboxylase Deficiency | PC (3534) | Hepatocytes, Astrocytes | Overexpression or off target expression could lead to metabolic imbalances, including overaccumulation of downstream metabolites | 12 |
| Spinocerebellar Ataxia 2; Parkinsons Disease | Atxn2 (3948) | Purkinje Cells, Basal Ganglia Neurons | Different cell populations can be targeted for different pathologies using the same transgene; Purkinje cells for SCA2, but to Basal Ganglia for PD | 13,14 |
| Spinocerebellar Ataxia 44 | Grm1 (3585) | Purkinje Cells | Some patients display both cerebellar and cortical involvement, but Grm1 is expressed at much higher levels in Purkinje cells. | 15,16 |
| SYNGAP1-related intellectual disability | SYNGAP1 (3879) | Hippocampal neurons, Cerebellar granule cells | SYNGAP1 is not expressed in non-neuronal cell types in the CNS, and its sequence length limits inclusion of additional regulatory elements in AAV genome. | 17 |
| Wilson's Disease | ATP7B (4410) | Hepatocytes, Basal Ganglia Neurons, Cornea | Hepatocytes express ATP7B at a much higher level than other tissues, thus enhancement may be desirable there while not overexpressing in other tissues | 18 |

**Supplementary Table 1 References**


1. Chiuchiolo, M. J. & Crystal, R. G. Gene Therapy for Alpha-1 Antitrypsin Deficiency Lung Disease. *Annals ATS* **13**, S352–S369 (2016).
2. Stoller, J. K., Hupertz, V. & Aboussouan, L. S. Alpha-1 Antitrypsin Deficiency. in *GeneReviews*® (eds. Adam, M. P. et al.) (University of Washington, Seattle, Seattle (WA), 1993).
3. Benke, T. A. *et al.* CDKL5 Deficiency Disorder. in *GeneReviews*® (eds. Adam, M. P. et al.) (University of Washington, Seattle, Seattle (WA), 1993).
4. Deardorff, M. A., Noon, S. E. & Krantz, I. D. Cornelia de Lange Syndrome. in *GeneReviews*® (eds. Adam, M. P. et al.) (University of Washington, Seattle, Seattle (WA), 1993).
5. Deciphering Developmental Disorders Study *et al.* BRD4 interacts with NIPBL and BRD4 is mutated in a Cornelia de Lange–like syndrome. *Nat Genet* **50**, 329–332 (2018).
6. Donati, B., Lorenzini, E. & Ciarrocchi, A. BRD4 and Cancer: going beyond transcriptional regulation. *Mol Cancer* **17**, 164 (2018).
7. Sarogni, P. *et al.* Overexpression of the cohesin-core subunit SMC1A contributes to colorectal cancer development. *J Exp Clin Cancer Res* **38**, 108 (2019).
8. Savant, A., Lyman, B., Bojanowski, C. & Upadia, J. Cystic Fibrosis. in *GeneReviews*® (eds. Adam, M. P. et al.) (University of Washington, Seattle, Seattle (WA), 1993).
9. Duan, D., Goemans, N., Takeda, S., Mercuri, E. & Aartsma-Rus, A. Duchenne muscular dystrophy. *Nat Rev Dis Primers* **7**, 13 (2021).
10. Steiner, R. D. & Basel, D. COL1A1/2 Osteogenesis Imperfecta. in *GeneReviews*® (eds. Adam, M. P. et al.) (University of Washington, Seattle, Seattle (WA), 1993).

11. Chen, Y. *et al.* Type-I collagen produced by distinct fibroblast lineages reveals specific function during embryogenesis and Osteogenesis Imperfecta. *Nat Commun* **12**, 7199 (2021).

12. Duque Lasio, M. L., Lehman, A. N., Ahmad, A. & Bedoyan, J. K. Pyruvate Carboxylase Deficiency. in *GeneReviews®* (eds. Adam, M. P. et al.) (University of Washington, Seattle, Seattle (WA), 1993).

13. Pulst, S. M. Spinocerebellar Ataxia Type 2. in *GeneReviews®* (eds. Adam, M. P. et al.) (University of Washington, Seattle, Seattle (WA), 1993).

14. Casse, F. *et al.* Detection of *ATXN2* Expansions in an Exome Dataset: An Underdiagnosed Cause of Parkinsonism. *Movement Disord Clin Pract* **10**, 664–669 (2023).

15. Perlman, S. Hereditary Ataxia Overview. in *GeneReviews®* (eds. Adam, M. P. et al.) (University of Washington, Seattle, Seattle (WA), 1993).

16. Watson, L. M. *et al.* Dominant Mutations in GRM1 Cause Spinocerebellar Ataxia Type 44. *The American Journal of Human Genetics* **101**, 451–458 (2017).

17. Holder, J. L., Hamdan, F. F. & Michaud, J. L. SYNGAP1-Related Intellectual Disability. in *GeneReviews®* (eds. Adam, M. P. et al.) (University of Washington, Seattle, Seattle (WA), 1993).

18. Weiss, K. H. & Schilsky, M. Wilson Disease. in *GeneReviews®* (eds. Adam, M. P. et al.) (University of Washington, Seattle, Seattle (WA), 1993).
