# Supplementary material for: Spatial genomics of AAV vectors reveals mechanism of transcriptional crosstalk that enables targeted delivery of large genetic cargo
Source: Nat Biotechnol. 2025 Mar 20;44(1):133–45. doi: 10.1038/s41587-025-02565-4 (PMC12807873; doi:10.1038/s41587-025-02565-4)
Supplement: Supplementary file 2 — Reporting Summary [file 41587_2025_2565_MOESM2_ESM.pdf]

nature portfolio

Corresponding author(s): Gradinaru, Viviana

Last updated by author(s): 01/16/2025

Reporting Summary

Nature Portfolio wishes to improve the reproducibility of the work that we publish. This form provides structure for consistency and transparency in reporting. For further information on Nature Portfolio policies, see our Editorial Policies and the Editorial Policy Checklist.

Statistics

For all statistical analyses, confirm that the following items are present in the figure legend, table legend, main text, or Methods section.

n/a	Confirmed	
☐	☒	The exact sample size (*n*) for each experimental group/condition, given as a discrete number and unit of measurement
☐	☒	A statement on whether measurements were taken from distinct samples or whether the same sample was measured repeatedly
☐	☒	The statistical test(s) used AND whether they are one- or two-sided<br>*Only common tests should be described solely by name; describe more complex techniques in the Methods section.*
☒	☐	A description of all covariates tested
☐	☒	A description of any assumptions or corrections, such as tests of normality and adjustment for multiple comparisons
☐	☒	A full description of the statistical parameters including central tendency (e.g. means) or other basic estimates (e.g. regression coefficient) AND variation (e.g. standard deviation) or associated estimates of uncertainty (e.g. confidence intervals)
☐	☒	For null hypothesis testing, the test statistic (e.g. *F*, *t*, *r*) with confidence intervals, effect sizes, degrees of freedom and *P* value noted<br>*Give P values as exact values whenever suitable.*
☒	☐	For Bayesian analysis, information on the choice of priors and Markov chain Monte Carlo settings
☒	☐	For hierarchical and complex designs, identification of the appropriate level for tests and full reporting of outcomes
☒	☐	Estimates of effect sizes (e.g. Cohen's *d*, Pearson's *r*), indicating how they were calculated

Our web collection on statistics for biologists contains articles on many of the points above.

Software and code

Policy information about availability of computer code

Data collection

The following commercial software was used for data collection.
Confocal micrographs: Zeiss Zen (black, v2.3).
Epifluorescence micrographs: Keyence Viewer software (v01.03.01.01)
Open field test data: Noldus Ethovision XT 17
Quantitative PCR: ThermoFisher Design and Analysis Software (v1.5.1)
Droplet PCR: BioRad QX Manager (v1.2)
EEG: Sirenia Acquisition (v2.2.12)

Data analysis	The following commercial and open-source software was used for data analysis.<br>Statistical analysis: GraphPad Prism (v10.0.3)<br>Initial processing of confocal micrographs: Zeiss Zen Blue (v2.5.75.0)<br>Analysis of quantitative PCR data: ThermoFisher Design and Analysis Software (v1.5.1)<br>Analysis of Droplet PCR: BioRad QX Manager (v1.2)<br>Quantification of fluorescence micrographs: ImageJ (FIJI Distribution, v1.54f)<br>Quantification of cell-segmented fluorescence micrographs: CellProfiler (v4.2.5)<br>Cell segmentation: CellPose (v2.2)<br>Automation of cell segmentation: Napari (v0.4.17)<br>Open field test analysis: Noldus Ethovision XT 17<br>Gait analysis: MouseWalker (developed in Mendes et al., BMC Biol 13:50; v20160807). Available from: https://github.com/MouseWalker/MouseWalker/tree/v1<br>Seizure detection from EEG: Sirenia Seizure Pre (v2.2.13)

For manuscripts utilizing custom algorithms or software that are central to the research but not yet described in published literature, software must be made available to editors and reviewers. We strongly encourage code deposition in a community repository (e.g. GitHub). See the Nature Portfolio guidelines for submitting code & software for further information.

Data

Policy information about availability of data

All manuscripts must include a data availability statement. This statement should provide the following information, where applicable:

- Accession codes, unique identifiers, or web links for publicly available datasets
- A description of any restrictions on data availability
- For clinical datasets or third party data, please ensure that the statement adheres to our policy

All sequences of primers, probes, sgRNAs, and other sequence elements are provided in Supplementary Table 2. Raw imaging datasets for all quantified data are deposited in the Brain Image Library. Data used to generate figures will be deposited at Zenodo. All other data that support the findings of this study are available from the corresponding authors upon reasonable request.

Research involving human participants, their data, or biological material

Policy information about studies with human participants or human data. See also policy information about sex, gender (identity/presentation), and sexual orientation and race, ethnicity and racism.

Reporting on sex and gender	N/A
Reporting on race, ethnicity, or other socially relevant groupings	N/A
Population characteristics	N/A
Recruitment	N/A
Ethics oversight	N/A

Note that full information on the approval of the study protocol must also be provided in the manuscript.

Field-specific reporting

Please select the one below that is the best fit for your research. If you are not sure, read the appropriate sections before making your selection.

☒ Life sciences ☐ Behavioural & social sciences ☐ Ecological, evolutionary & environmental sciences

For a reference copy of the document with all sections, see nature.com/documents/nr-reporting-summary-flat.pdf

Life sciences study design

All studies must disclose on these points even when the disclosure is negative.

Sample size	No sample size calculations were performed. Animal sample sizes were chosen based on pilot experiment data and by comparison to similar published literature. Numbers of cells used in analyses were determined by automated segmentation of fluorescence micrographs.
Data exclusions	No data were excluded from the analyses, except for gait analysis trials in which paw or body tracking was determined to be inaccurate by a blinded experimenter.
Replication	All primary neuron experiments were repeated at least 2 times with similar results (i.e. 2 separate preparations of primary neurons, taken from separate dams). Similarly, all experiments in cell lines were repeated at least 2 times with similar results. All mouse experiments were

	conducted with 2-6 animals with similar results for animals in the same experimental conditions. For mouse experiments in Figs 5 and 6, pilot cohorts were used to assess feasibility of approach, and yielded results consistent with cohorts used in final analysis.
Randomization	All mice were randomly assigned to experimental conditions. For imaging, fields of view were selected using non-experimental channels (e.g. Hoechst, Nissl, or autofluorescence).
Blinding	For imaging, blinding was not necessary as we chose fields of view using non-experimental channels. For manual segmentation of cells or tissue regions, experimenters were blinded to experimental condition. For automated segmentation and analysis of segmented cells, experimenters were not blinded as bias is unlikely to be introduced during these steps.<br>For behavioural data acquisition and scoring, all researchers except G.M.C. were blinded to experimental assignment. To mitigate bias from this, G.M.C. only conducted narrowing beam behaviour and open field tests, and did not score or quantify any behavioural tests. Blinded experimenters or automated software quantified and scored all behavioural assays. However, we acknowledge that given the severity of behavioural phenotypes observed with Cacna1a disruption, even blinded experimenters would be able determine which animals were injected with AAVs to disrupt Cacna1a. EEG data collection was performed by blinding experimenters, and EEG data was analyzed with automated scoring software.

Reporting for specific materials, systems and methods

We require information from authors about some types of materials, experimental systems and methods used in many studies. Here, indicate whether each material, system or method listed is relevant to your study. If you are not sure if a list item applies to your research, read the appropriate section before selecting a response.

Materials & experimental systems

n/a	Involved in the study
☐	☒ Antibodies
☐	☒ Eukaryotic cell lines
☒	☐ Palaeontology and archaeology
☐	☒ Animals and other organisms
☒	☐ Clinical data
☒	☐ Dual use research of concern
☒	☐ Plants

Methods

n/a	Involved in the study
☒	☐ ChIP-seq
☒	☐ Flow cytometry
☒	☐ MRI-based neuroimaging

Antibodies

Antibodies used	Rabbit pAb against Cacna1a: Alomone labs ACC-001, lot #AN1902, RRID:AB_2039764<br>Chicken pAb against GFP: Aves #1020, validated by manufacturer and commonly used for IHC against GFP and derivatives. Lot: GFP3717982, RRID:AB_10000240<br>Rabbit pAb against TagRFP (used for detection of mRuby): Dr. Dawen Cai, University of Michigan, distributed through Cancer Tools #155266, validated by Cai et al., Nat Methods 10: 540. Lot: 2017B3, RRID:AB_3107169<br>Alexa Fluor 488 conjugated mouse mAb against AAV VP1/VP2/VP3: Clone B1, Progen, #61058-488, RRID:AB_3107170
Validation	Alomone labs ACC-001, validated by manufacturer and commonly used for IHC against Cacna1a (including in knockout studies in mice, e.g. Jung et al., Front. Behav. Neurosci 10: 214)<br>Aves #1020, validated by manufacturer and commonly used for IHC against GFP and derivatives.<br>Cancer Tools #155266, validated by Cai et al., Nat Methods 10: 540.<br>Progen #61058-488, B1 clone validated by Wistuba et al., J Virol 71: 1341

Eukaryotic cell lines

Policy information about cell lines and Sex and Gender in Research

Cell line source(s)	HEK293T cells (ATCC, CRL-3216, RRID:CVCL_0063)
Authentication	None of the used cell lines were authenticated, though normal HEK293T cell morphology was observed throughout experiments.
Mycoplasma contamination	Stocks of HEK293T cells were tested for mycoplasm contamination and confirmed negative, using ATCC 30-1012K.
Commonly misidentified lines<br>(See ICLAC register)	None of the lines used are listed on the ICLAC register

Animals and other research organisms

Policy information about studies involving animals; ARRIVE guidelines recommended for reporting animal research, and Sex and Gender in Research

Laboratory animals	The following mice were used:

Laboratory animals	8--week old wildtype C57BL/6J mice (Jackson Laboratory, strain #000664, RRID:IMSR_JAX:000664)<br>8-week old C57BL/6J-background Prkdc scid/scid (Jackson Laboratory, strain #: 001913, RRID:IMSR_JAX:001913)<br>8-week old C57BL/6J-background Rosa26CAG-LSL-tdTomato (Jackson Laboratory, strain #: 007914, RRID:IMSR_JAX:007914)<br>For primary neuronal cultures: Timed pregnant C57BL/6N (Charles River, RRID:MGI:2159965)
Wild animals	No wild animals were used in the study
Reporting on sex	For experiments in which adult mice were the biological unit, only male mice were used.
Field-collected samples	The study did not involve any field-collected samples
Ethics oversight	For mouse experiments, animal husbandry and all procedures involving animals were performed in accordance with the Guide for the Care and Use of Laboratory Animals of the National Institutes of Health and approved by the Institutional Animal Care and Use Committee (IACUC) and by the Office of Laboratory Animal Resources at the California Institute of Technology.

Note that full information on the approval of the study protocol must also be provided in the manuscript.

Plants

Seed stocks	N/A
Novel plant genotypes	N/A
Authentication	N/A
